# Distinct surveillance pathway for immunopathology during acute infection via autophagy and SR-BI

**DOI:** 10.1038/srep34440

**Published:** 2016-10-03

**Authors:** Susanne Pfeiler, Avinash B. Khandagale, Astrid Magenau, Maryana Nichols, Harry F. G. Heijnen, Franz Rinninger, Tilman Ziegler, Stephanie Seveau, Sören Schubert, Stefan Zahler, Admar Verschoor, Eicke Latz, Steffen Massberg, Katharina Gaus, Bernd Engelmann

**Affiliations:** 1 Institut für Laboratoriumsmedizin, Ludwig-Maximilians-Universität, Munich, 81377, Germany; 2 Centre for Vascular Research, ARC Centre for Excellence in Advanced Molecular Imaging and Australian Centre for Nanomedicine, University of New South Wales, Sydney, New South Wales 2052, Australia; 3 Laboratory of Clinical Chemistry and Haematology and Cell Microscopy Center, University Medical Center Utrecht, Utrecht, 3584CX, The Netherlands; 4 Universitätsklinik Hamburg-Eppendorf, Hamburg, 20246, Germany; 5 Medizinische Klinik und Poliklinik I, Ludwig-Maximilians-Universität, Munich, 81377, Germany; 6 Department of Microbiology, The Ohio State University, Columbus, OH 43210, USA; 7 Max von Pettenkofer-Institut, Ludwig-Maximilians-Universität, Munich, 80336, Germany; 8 Institut für Pharmazeutische Biologie, Ludwig-Maximilians-Universität, Munich, 81377, Germany; 9 Institut für Systemische Entzündungsforschung, Universität zu Lübeck, Lübeck, 23538, Germany; 10 Institute of Innate Immunity, University of Bonn, Bonn, 53127, Germany; 11 Department of Infectious Diseases and Immunology, UMass Medical School, Worcester, MA 01605, USA; 12 German Center for Neurodegenerative Diseases (DZNE), Bonn, 53127, Germany

## Abstract

The mechanisms protecting from immunopathology during acute bacterial infections are incompletely known. We found that in response to apoptotic immune cells and live or dead *Listeria monocytogenes* scavenger receptor BI (SR-BI), an anti-atherogenic lipid exchange mediator, activated internalization mechanisms with characteristics of macropinocytosis and, assisted by Golgi fragmentation, initiated autophagic responses. This was supported by scavenger receptor-induced local increases in membrane cholesterol concentrations which generated lipid domains particularly in cell extensions and the Golgi. SR-BI was a key driver of beclin-1-dependent autophagy during acute bacterial infection of the liver and spleen. Autophagy regulated tissue infiltration of neutrophils, suppressed accumulation of Ly6C^+^ (inflammatory) macrophages, and prevented hepatocyte necrosis in the core of infectious foci. Perifocal levels of Ly6C^+^ macrophages and Ly6C^−^ macrophages were unaffected, indicating predominant regulation of the focus core. SR-BI-triggered autophagy promoted co-elimination of apoptotic immune cells and dead bacteria but barely influenced bacterial sequestration and survival or inflammasome activation, thus exclusively counteracting damage inflicted by immune responses. Hence, SR-BI- and autophagy promote a surveillance pathway that partially responds to products of antimicrobial defenses and selectively prevents immunity-induced damage during acute infection. Our findings suggest that control of infection-associated immunopathology can be based on a unified defense operation.

The collateral host damage induced by immune responses during infections and sterile injuries represents a major cost of immunity. Such collateral damage, or immunopathology, is observed in a variety of species ranging from plants and arthropods to mammals^1^,^2^. Microbicidal and cytotoxic immune cells are major inducers of immunopathology as exemplified by liver tissue deterioration by infiltrating innate immune cells^3^ or irreversibly damaged bronchi caused by CD8^+^ T cells^4^. Host injury by immune responses as for example during viral infection is promoted in particular by the excessive formation of proinflammatory cytokines^5^. Furthermore, in addition to live immune cells also apoptotic immune cells can induce immunopathology such as by triggering auto-immune reactions^6^.

Mammalian hosts have evolved various mechanisms to prevent host damage by immune responses in particular during chronic infections. These include for example type I interferons (IFN), which can protect from collateral host damage during viral infections of the respiratory system^7^, and virus-specific CD8^+^ T cells that reduce immunopathology by virus-specific CD4^+^ T cells in mice infected with lymphocytic choriomeningitis virus^8^. Moreover, detrimental effects of immune reactions on host integrity are switched off by resolution of inflammation, which is supported by specialized resolution mediators as well as anti-inflammatory cytokines^9^, and critically involves microbicidal activities^10^,^11^. Thus, there is increasing knowledge about how suppression of immunity-induced tissue injury during viral infections as well as termination of inflammation protect from immunopathology. Instead, it is less well understood how hosts balance protection from tissue injury by immune responses and pathogen control during acute bacterial infections.

Here we show that in host organs infected with *Listeria monocytogenes* (or *Yersinia enterocolitica*) beclin-1-mediated autophagic responses are activated by SR-BI to protect organs such as the liver and spleen selectively from the collateral damage induced by antibacterial defenses. We identified this scavenger receptor, which promotes lipid exchange between lipoproteins and cells^12^,^13^ and is involved in host cell-pathogen interactions^14^,^15^, as a regulator of lipid domain formation that in turn increased cellular uptake mechanisms with features of macropinocytosis and promoted autophagic flux. Beclin-1-driven autophagy suppressed tissue damage by antimicrobial defenses in infected organs *in vivo* without affecting sequestration or survival of tissue-associated bacteria. It prevented formation of necrotic cells in the core of infectious foci, promoted clearance of apoptotic immune cells, and decreased tissue infiltration and accumulation of neutrophils and inflammatory macrophages. Our study thus reveals autophagy as a chief protector from immunity-caused organ damage during acute infection. Overall, cooperations between SR-BI and autophagic responses are suggested to promote an independent defense process that can be triggered by products of antimicrobial reactions such as apoptotic immune cells and distinctively controls immunopathology.

## Results

### SR-BI prevents organ damage during bacterial infections without affecting bacterial containment and survival

The liver is a major organ of pathogen colonization^16^ and a central hub of cholesterol metabolism which may be implicated in immune functions^17^. At day 3 (d3) of infection with *Listeria monocytogenes* (10^5^ cfu), when bacterial colonization of liver and caspase-1-mediated microbicidal activities are at a maximum^18^, liver cholesterol contents were increased compared to d0. Concomitantly, blood plasma cholesterol levels were decreased (Supplementary Fig. 1a), which together suggested that liver cholesterol supply was enhanced. Cholesterol transfer to the liver is supported by LDL receptor (LDLR), SR-BI, and low density related protein 1 (LRP1)^19^.

**Figure 1:**
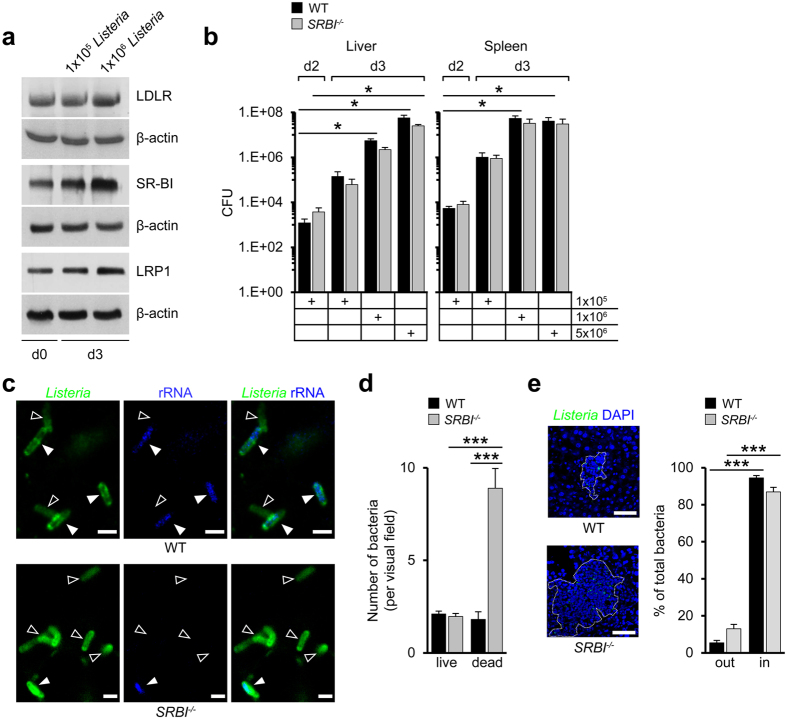
Scavenger receptor-BI prevents tissue damage without altering pathogen burden. (**a**) Expression of liver cholesterol transporters in *Listeria*-infected WT mice. Representative western blots on three different mice. Superscripts refer to pathogen loads. (**b**) Bacterial survival in *Listeria*-infected organs of WT and *SRBI*^−/−^ mice. Animals infected with different pathogen loads (cfu) as indicated. n = 3 (5 × 10^6^), n = 5 (1 × 10^6^), n = 6 (1 × 10^5^, d2; WT, spleen, d3), n = 7 (1 × 10^5^, d3, *SRBI*^−/−^), n = 8 (1 × 10^5^, d2, WT, liver). (**c**) Confocal microscopy images of rRNA-positive (full arrowheads; blue, pseudocolored) and rRNA-negative bacteria (empty arrowheads) in the livers of mice infected with *Listeria* (10^5^) representing live and dead bacteria, respectively (d3). Scale bar, 2 μm. (**d**) Quantification of tissue-associated rRNA-positive and rRNA-negative *Listeria* (d3). n = 3, ten images per animal. (**e**) Localization of *Listeria* within infectious foci. Left: Confocal microscopy images. Dotted line indicate focus boundary. Scale bar, 50 μm. Right: *Listeria* distribution inside and outside of infectious foci. Values expressed as percentage of total bacteria (d3). n = 3, five images per animal. ***P < 0.001. Kruskal-Wallis One Way Analysis of Variants on Ranks (**b**) and One-way ANOVA with Bonferroni post-test correction (**d**,**e**). Data are representative of experiments on three different mice (**a**,**c**,**e**).

Liver LDLR expression was unchanged during *Listeria* infection while expressions of SR-BI and LRP1 were markedly and slightly enhanced, respectively (Fig. 1a, Supplementary Fig. 1b). During infection with *Yersinia enterocolitica* (5 × 10^6 ^cfu) we observed similar changes in SR-BI expression and in cholesterol levels as in *Listeria*-infected mice (Supplementary Fig. 1c,d). In contrast to WT mice, *Listeria* infection increased plasma cholesterol levels in mice deficient for SR-BI (*SRBI*^−/−^) (from 149 ± 17 (d0, n = 3) to 223 ± 13 mg/dl (d3, n = 5), P < 0.05). Next, we visualized the cholesterol localization in the liver with filipin. *Listeria* infection augmented cholesterol levels at the plasma membrane of hepatocytes in WT mice (Supplementary Fig. 1e) but not in *SRBI*^−/−^ mice. Pathogen burden in the liver and spleen of WT mice increased between d2 and d3 (Fig. 1b) without further increasing at d4 (*data not shown*). Pathogen burden increased to a similar extent in *SRBI*^−/−^ mice as in WT mice at all pathogen loads investigated (Fig. 1b). Also survival of *Yersinia* was comparable in WT and *SRBI*^−/−^ mice (Supplementary Fig. 1f).

Nonetheless, visualization of *Listeria* indicated that the number of liver-associated bacteria in *SRBI*^−/−^ mice was higher than in WT mice (Fig. 1c,e). To examine whether part of the labeled bacteria represented dead bacteria, we hybridized the tissues with oligonucleotides complementary to *Listeria*-rRNA, given that bacterial rRNA is rapidly degraded in dead but not live bacteria^20^. Most bacteria in *SRBI*^−/−^ mice were rRNA-negative. In WT mice, almost equal amounts of rRNA-positive and -negative bacteria were seen at d3 (Fig. 1c,d). The presence of dead bacteria at levels equalling (WT) or exceeding (*SRBI*^−/−^) live bacteria indicated robust microbicidal activities at d3 in both types of mice. Together with the observation of a comparable number of live bacteria in WT and *SRBI*^−/−^ mice this suggested that the increase in dead bacteria in *SRBI*^−/−^ mice was due their impaired elimination.

Almost all dead and live bacteria were sequestered inside of tissue-associated infectious foci in WT and *SRBI*^−/−^ mice (Fig. 1e), consistent with the role of foci as sites of bacterial killing^21^. Thus, bacterial sequestration was not affected by SR-BI. However, the area of infectious foci was substantially increased in *Listeria*-infected *SRBI*^−/−^ mice (Supplementary Fig. 1g). Foci contained higher numbers of bacteria than in WT animals due to the increase in dead bacteria. Foci were also markedly enlarged in the kidneys of *Listeria*-infected *SRBI*^−/−^ mice as well as in the liver, spleen and kidney of *Yersinia*-infected *SRBI*^−/−^ mice (Supplementary Fig. 1h,i). Since foci displaced substantially more normal liver tissue in *SRBI*^−/−^ mice than in WT mice (Supplementary Fig. 1g) we investigated whether their increased growth was associated with hepatocyte injury.

Necrotic HNF4α^+^ hepatocytes as detected by their pyknotic nuclei and eosinophilic cytoplasm were almost exclusively localized inside the core of foci (Supplementary Fig. 1j). In both, WT and *SRBI*^−/−^ mice, most of the necrotic cells detected represented hepatocytes. Such necrotic hepatocytes were markedly increased in *SRBI*^−/−^ relative to WT mice. Also, ALT values in blood, an indirect measure of hepatocyte necrosis and thus of tissue damage, were higher in *SRBI*^−/−^ than in WT animals during infection with *Listeria* (Supplementary Fig. 2a). When we plotted ALT values as a function of organ pathogen loads, the curve relating pathogen load to tissue damage only weakly increased in WT mice while it steeply increased in *SRBI*^−/−^ mice (Supplementary Fig. 2a), indicating that the scavenger receptor prevented necrotic tissue injury at different levels of bacterial burden.

### Reduction of tissue accumulation of neutrophils and inflammatory macrophages via SR-BI

**Figure 2:**
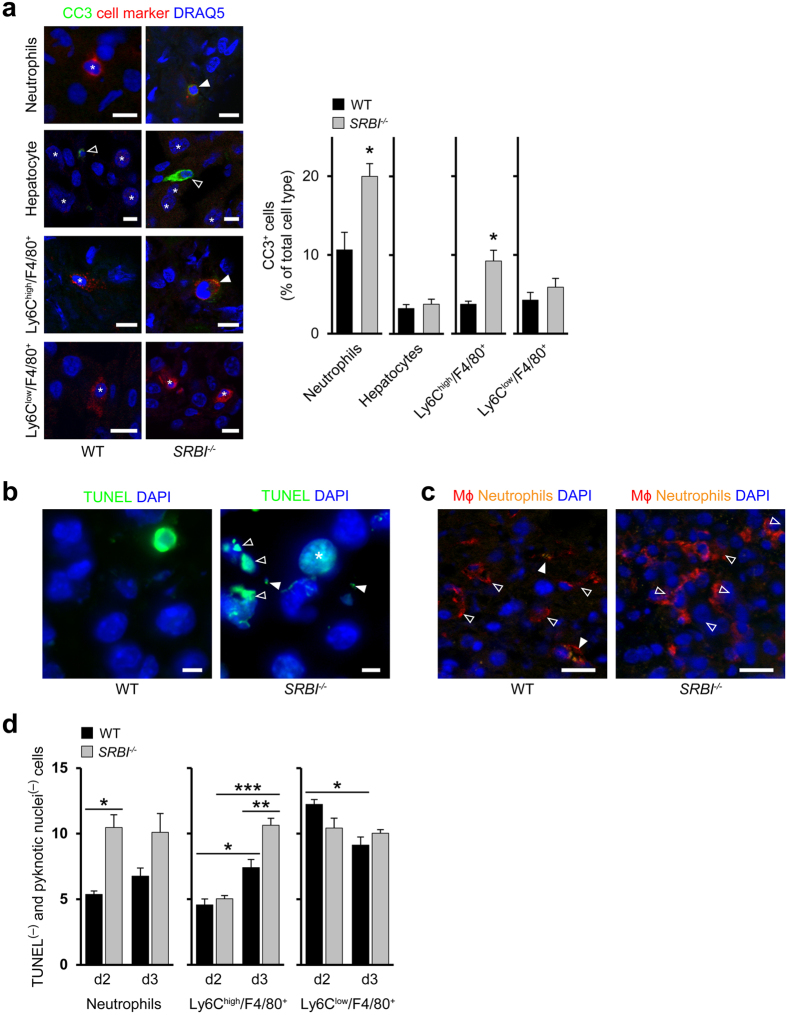
SR-BI suppresses tissue accumulation of neutrophils and inflammatory macrophages. (**a**) Visualization of cell type-specific apoptosis. Left: Confocal microscopy images: Full arrowheads, apoptotic cell (colocalization of cell specific marker and CC3^+^); empty arrowheads, CC3^+^ cell; star, cell specific marker; Ly6C^high^F4/80^+^, inflammatory macrophages (originating from inflammatory monocytes); Ly6C^low^F4/80^+^, macrophages lacking signs of inflammatory monocytes. DRAQ5, nuclei marker (blue, pseudocolored), Scale bar, 10 μm. Right: Quantification of cells positive for CC3^+^ (d3) as percentage of total cell type. n = 3, five images per animal. (**b**) Apoptotic bodies and apoptotic cells with fragmented nuclei during *Listeria* infection (d3). Confocal microscopy images: Empty arrowheads, apoptotic cells with fragmented nuclei; full arrowheads, apoptotic bodies; stars, apoptotic cells. Scale bar, 10 μm. (**c**) Macrophages with internalized Ly6G^+^ neutrophils in the livers of *Listeria* infected mice (d3). Confocal microscopy images: Empty arrowheads, tissue macrophages; filled arrowheads, tissue macrophages with internalized neutrophils. Scale bar, 20 μm. (**d**) Number of cells lacking signs of cell death (cell marker^+^, TUNEL^−^. normal-sized nuclei) per visual field in *Listeria*-infected WT and *SRBI*^−/−^ mice. n = 3, ten images per animal. *P < 0.05, **P < 0.01, ***P < 0.001. *t*-test (**a**) and One-way ANOVA with Bonferroni post-test correction (**d**). Data are representative of experiments on three different mice (**a**–**c**).

Since innate immune cells are major causes of tissue damage^1^,^2^,^3^ we investigated whether SR-BI elicited changes in their tissue infiltration. Ly6G^+^ neutrophils were augmented in *Listeria*-infected livers of *SRBI*^−/−^ mice relative to WT mice, while levels of total macrophages were comparable (Supplementary Fig. 2b,c). Consistent with neutrophil apoptosis during inflammatory responses^6^, the percentages of tissue-associated neutrophils positive for apoptosis marker cleaved caspase-3 (CC3^+^) were increased in *SRBI*^−/−^ animals compared to WT animals (Fig. 2a). The percentages of CC3^+^ Ly6C^high^ macrophages, which are derived from inflammatory monocytes^22^, were also augmented. Instead, CC3^+^ hepatocytes and CC3^+^ Ly6C^low^ macrophages were unchanged in *SRBI*^−/−^ compared to WT animals (Fig. 2a). The amount of TUNEL^+^ cells, which apart from apoptotic cells also include necroptotic cells^23^, was enhanced in the liver and spleen of *Listeria*- and *Yersinia* infected *SRBI*^−/−^ relative to WT animals (Supplementary Fig. 2d,e). Such TUNEL^+^ cells almost exclusively accumulated in infectious foci (Supplementary Fig. 2f).

In *SRBI*^−/−^ mice, both the area occupied by dead host cells (identified as TUNEL^+^ cells and/or cells with pyknotic or fragmented nuclei) and the percentage of total area covered by dead host cells were augmented compared to WT mice (Supplementary Fig. 2f). Most of the TUNEL^+^ cells in WT and *SRBI*^−/−^ animals represented neutrophils, hepatocytes and macrophages. TUNEL^+^ neutrophils and macrophages increased more strongly in *SRBI*^−/−^ animals compared to WT mice than TUNEL^+^ hepatocytes so that the contribution of professional immune cells to total TUNEL^+^ cells became even stronger (Supplementary Fig. 2g). Overall, TUNEL^+^ innate immune cells mostly contributed to the rise in TUNEL^+^ cells and enhanced focus area in *SRBI*^−/−^ mice.

We detected substantial amounts of apoptotic bodies in the livers of *SRBI*^−/−^ mice but not in WT mice (Fig. 2b). Since apoptotic bodies are signs of late apoptosis, their persistence indicated a delayed clearance of apoptotic cells. In WT mice, in contrast to *SRBI*^−/−^ mice, several macrophages contained ingested neutrophils (Fig. 2c), suggesting that SR-BI enhanced engulfment of (apoptotic) neutrophils. The increased accumulation of neutrophils and macrophages in *SRBI*^−/−^ mice (Supplementary Fig. 2b,c), apart from being caused by a decreased clearance of dead immune cells, could additionally result from an enhanced tissue infiltration of immune cells. Indeed, the number of live neutrophils and live Ly6C^high^ macrophages were higher in *SRBI*^−/−^ compared to WT mice (Fig. 2d). Thus, SR-BI promoted removal of apoptotic immune cells, inhibited extravasation of neutrophils and prevented tissue accumulation of live inflammatory macrophages.

### SR-BI enhances membrane cholesterol concentrations at specific subcellular sites

We next analyzed at the cellular level how SR-BI supported organ protection. We hypothesized that the ability of SR-BI to control cell cholesterol *in vivo* (Supplementary Fig. 1e) contributed to its protective functions. To detect membrane regions with increased cholesterol concentrations we performed immunoelectron microscopy with a perfringolysin O (PFO) derivative (BCΘ^24^). In CHO cells transfected with human SR-BI (hSR-BI^+^) the cholesterol concentrations were augmented in plasma membrane ruffles and along the endocytic pathway (Supplementary Fig. 3a). SR-BI itself co-localized with these areas (Supplementary Fig. 3a,b), suggesting that SR-BI accumulated cholesterol in its vicinity. The scavenger receptor induced similar changes in the cell periphery of human macrophages since the local BCΘ-dependent fluorescence was increased in wt macrophages (SR-BI_wt_ macrophages) compared to macrophages with reduced SR-BI expression (using siRNA; SR-BI_red_ macrophages) (Supplementary Fig. 3c). The changes in membrane cholesterol concentrations did not alter the number of lipid bodies (Supplementary Fig. 3d), intracellular reservoirs of cholesterol that participate in inflammatory responses^25^.

**Figure 3:**
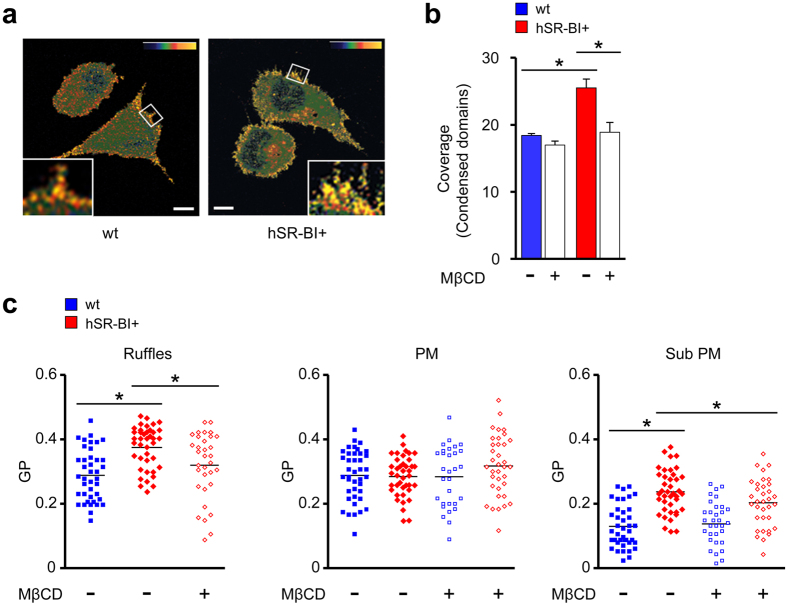
Subcellular site-specific changes in membrane cholesterol levels via SR-BI. (**a**) SR-BI induces changes in structure of cellular membranes. Representative pictures of Laurdan fluorescence of wt and hSR-BI+cells converted into pseudocolored images of generalized polarization (GP) values. Upper right corner: scale for GP values. Minimum and maximum values of color bar: −1 (blue) and +1 (yellow). Small boxes indicate area magnified in large boxes. Scale bar, 5 μm. Data are representative of three different experiments. (**b**) Area covered by ordered domains in wt and hSR-BI+ cells. Where indicated, cells were preincubated with MβCD. 20–30 cells per cell type. (**c**) GP values at ruffles, plasma membrane (PM) and submembraneous areas (Sub PM). Wt and hSR-BI+ cells without and with MβCD treatment. In hSR-BI+ cells no ruffles could be detected after MβCD treatment. Each symbol represents a single cell; small horizontal lines indicate the mean. *P < 0.05. Kruskal-Wallis One Way Analysis of Variants on Ranks (**b**,**c**).

The fluorescent probe Laurdan indicates the presence of ordered membrane domains originating from local cholesterol enrichment^26^. The area of ordered domains was increased in hSR-BI^+^ cells compared to wt cells (Fig. 3a,b). This increase was prevented by reducing cholesterol with methyl-β-cyclodextrin (MβCD) (Fig. 3b) which showed that the ordered membranes indeed represented cholesterol-enriched domains. Underscoring the results obtained with BCΘ, SR-BI elevated membrane order in cell membrane ruffles and in submembraneous regions but not in the bulk of the plasma membrane (Fig. 3c). Next, we tested the CDSRCD and SRCDSR mutants of murine SR-BI^27^ (mSR-BI), which contain only the extracellular domain and the transmembrane domain of SR-BI, respectively. Similar to hSR-BI, native mSR-BI enhanced membrane order in ruffles and submembraneous regions (Supplementary Fig. 3e). The CDSRCD and SRCDSR mutants increased membrane order less efficiently at these sites than native mSR-BI. Overall, SR-BI increased membrane cholesterol concentrations in the cell periphery via both its extracellular and transmembrane portions. Cholesterol domain formation in the submembraneous region might require the extracellular and transmembrane portions of SR-BI for different reasons such as for example to enhance internalization of cholesterol and to accumulate the lipid in its vicinity, respectively. On the other hand, domain formation in ruffles might depend on a cooperation of both portions of the scavenger receptor.

### Internalization of bacteria and apoptotic cells via regulation of cellular cholesterol levels

**Figure 4:**
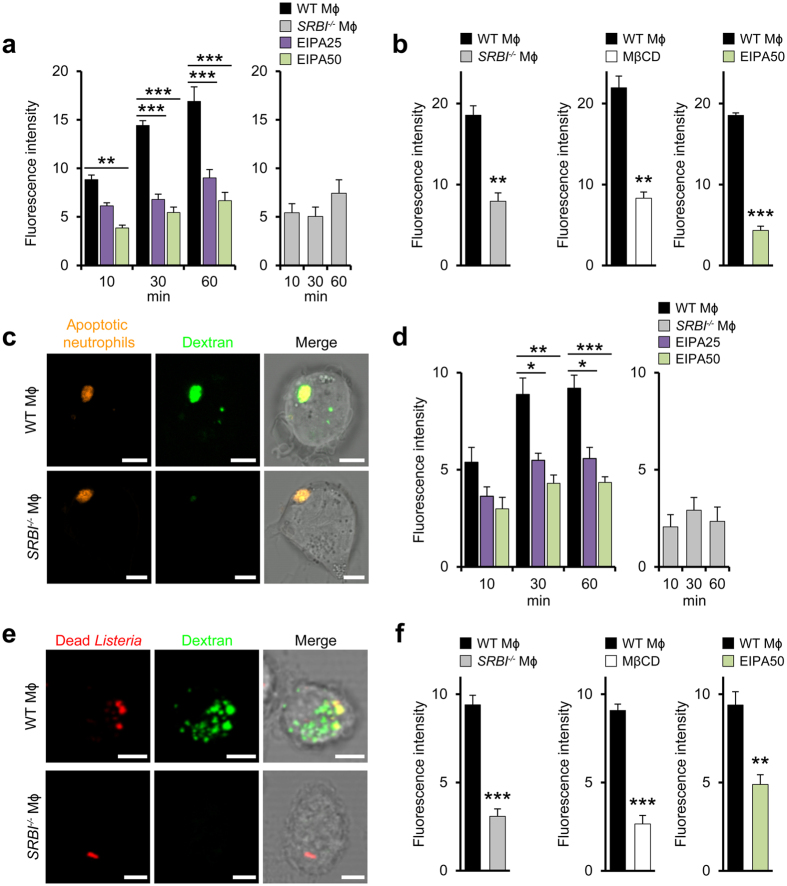
Local increases in membrane cholesterol levels regulate macropinocytic uptake of apoptotic cells and bacteria. (**a**) SR-BI-dependent uptake of macropinocytosis marker dextran. Internalization of 70 kDa dextran by WT (left) and *SRBI*^−/−^ macrophages (right). Effect of EIPA (μM). n = 3, ten images per experiment. (**b**) SR-BI-induced engulfment of labeled apoptotic neutrophils by WT and *SRBI*^−/−^ macrophages (left). Influence of MβCD and EIPA (μM). The fluoresence intensity refers to the internalized apoptotic cells. n = 3, ten images per experiment. (**c**) Colocalization of engulfed apoptotic neutrophils with dextran in macrophages. Confocal microscopy images. Scale bar, 5 μm. (**d**) Uptake of dead *Listeria* by WT (left) and *SRBI*^−/−^ macrophages (right). Effect of EIPA (μM). n = 3, ten images per experiment. (**e**) Colocalization of incorporated dead bacteria with dextran in macrophages. Confocal microscopy images. Scale bar, 5 μm. (**f**) Influence of SR-BI on engulfment of labeled *Listeria* by macrophages (left). Influence of MβCD and EIPA (μM). n = 3 and n = 6 (middle panel), ten images per experiment. *P < 0.05, **P < 0.01, ***P < 0.001. One-way ANOVA with Bonferroni post-test correction (**a**,**d**), *t*-test (**b**,**f**). Data are representative of three different experiments (**c**,**e**).

Local increases in membrane cholesterol concentrations could activate macropinocytosis since this endocytic pathway requires membrane ruffles and is enhanced by ruffle-associated cholesterol^28^. Macrophages isolated from WT mice (WT macrophages) internalized dextran, a marker for macropinocytic internalization, more rapidly than *SRBI*^−/−^ macrophages (Fig. 4a). Dextran uptake was inhibited by EIPA, a specific inhibitor of macropinocytosis^29^ (Fig. 4a). Given that SR-BI enhanced neutrophil engulfment by macrophages *in vivo* (Fig. 2c), we examined whether macropinocytosis participated in cell entry of apoptotic cells. SR-BI increased engulfment of apoptotic neutrophils by mouse and human macrophages (Fig. 4b, Supplementary Fig. 4a; ref. 30) which was prevented in part by MβCD and EIPA. In WT macrophages, in contrast to *SRBI*^−/−^ macrophages, the engulfed apoptotic cells accumulated inside dextran-containing intracellular compartments (Fig. 4c).

SR-BI also increased uptake of dead (heat-inactivated) bacteria (Fig. 4d) and moreover enhanced internalization of *Listeria* (Fig. 4f). EIPA partially inhibited internalization of dead *Listeria* in WT macrophages. Furthermore, in WT macrophages, but not in *SRBI*^−/−^ macrophages, the internalized bacterial corpses co-localized with internalized dextran (Fig. 4e). Also cell entry of *Listeria* into WT macrophages was suppressed in part by EIPA (Fig. 4f) and, extending previous observations^31^, by MβCD. The internalized bacteria co-associated with dextran in WT macrophages but not in *SRBI*^−/−^ macrophages (Supplementary Fig. 4b).

Caveolin-1 (Cav-1) induces formation of caveolae^32^, membrane domains accumulating cholesterol that predominate in the cell periphery and thus exhibit partially similar characteristics as SR-BI-mediated cholesterol clustering. WT macrophages internalized more apoptotic neutrophils than *Cav-1*^−/−^ macrophages (Supplementary Fig. 4c) which was inhibited by MβCD. In contrast, inhibition of the formation of lipid bodies with C75^33^ did not affect macrophage engulfment of apoptotic cells (Supplementary Fig. 4d). Thus, SR-BI-induced increases in membrane cholesterol levels promoted macrophage uptake of pathogens and apoptotic neutrophils which involved macropinocytosis.

### Scavenger-receptor-induced elimination of host cell and bacterial corpses via autophagy

**Figure 5:**
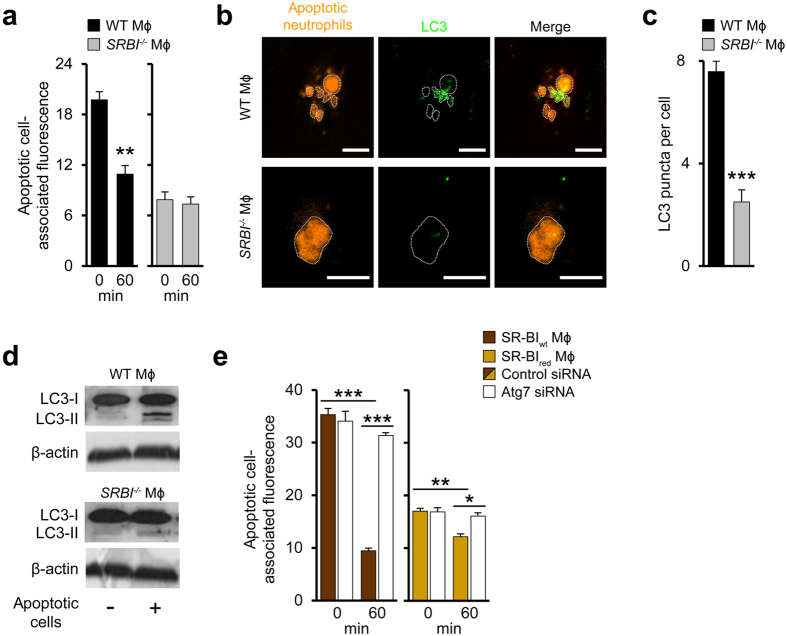
Degradation of host cell and bacterial corpses by scavenger receptor-triggered autophagy. (**a**) Degradation of engulfed apoptotic cells in WT (left) and *SRBI*^−/−^ (right) macrophages. Changes in fluorescence intensity associated with engulfed apoptotic neutrophils. 0 min refers to time point after 30 min engulfment period. n = 3, ten images per experiment. (**b**) LC3 association with engulfed apoptotic cells in macrophages. Confocal microscopy images: Apoptotic cell structures delineated with dotted lines. Scale bar, 5 μm. Data are representative of three independent experiments. (**c**) LC3 puncta in macrophages incubated with apoptotic neutrophils. n = 6, ten images per experiment. (**d**) LC3-II formation in macrophages incubated with apoptotic neutrophils. Representative western blots from three different experiments. (**e**) Degradation of apoptotic neutrophils in SR-BI_wt_ and SR-BI_red_ macrophages without or with reduced Atg7 expression. Changes in fluorescence intensity associated with engulfed apoptotic neutrophils. 0 min refers to time point after 30 min engulfment period. n = 3, ten images per experiment. *P < 0.05, **P < 0.01, ***P < 0.001. *t*-test (**a**,**c**) and One-way ANOVA with Bonferroni post-test correction (**e**).

TAMRA-labelled apoptotic neutrophils engulfed by macrophages were rapidly degraded in WT but not in *SRBI*^−/−^ macrophages (Fig. 5a). Degradation of apoptotic cells can be facilitated by (macro)autophagy^34^. The engulfed apoptotic cells were decorated with the essential autophagy protein LC3 in WT macrophages but not in *SRBI*^−/−^ macrophages (Fig. 5b). Also, LC3 recruitment to autophagic vesicles as measured by LC3 puncta was substantially higher in WT macrophages relative to *SRBI*^−/−^ macrophages (Fig. 5c). Following cell corpse engulfment, LC3-II is formed during autophagy induction by lipidation of LC3-I^35^. Following exposure to apoptotic cells, LC3-II formation was enhanced in WT macrophages but not in *SRBI*^−/−^ macrophages (Fig. 5d).

Next, we suppressed expression of Atg7 via siRNA, a central autophagy mediator catalyzing activation of Atg12^36^ (Supplementary Fig. 4e). This inhibited the increase in LC3 puncta induced by SR-BI in human macrophages exposed to apoptotic neutrophils (Supplementary Fig. 4f). Atg7 inhibition prevented degradation of apoptotic neutrophils in SR-BI_wt_ macrophages and reduced the limited cell corpse degradation in SR-BI_red_ macrophages (Fig. 5e). Also pharmacological inhibition of autophagic flux by abrogating phosphatidylinositol-3-kinase activity with wortmannin or 3-MA prevented SR-BI-dependent elimination of apoptotic cells (Supplementary Fig. 4g,h). Moreover, autophagy induction by SR-BI supported degradation of dead *Listeria* in human and mouse macrophages (Supplementary Fig. 4i,j).

Also dead bacteria as well as live bacteria^37^ triggered autophagy in a SR-BI-dependent way as indicated by the lower number of LC3 puncta and a decreased LC3-II formation in macrophages with low SR-BI expression or *SRBI*^−/−^ macrophages (Supplementary Fig. 5a–d). The effect of SR-BI on LC3 puncta was somewhat less pronounced than with the autophagy activator rapamycin. Autophagy activation in *Listeria*-infected macrophages was abrogated by MβCD (Supplementary Fig. 5d). Furthermore, SR-BI also triggered autophagic responses in *Listeria*- and *Yersinia*-infected CHO cells (Supplementary Fig. 5e,f). Despite efficient autophagy activation, viability of *Listeria* remained unaffected by changes of SR-BI expression in different cell types (Supplementary Fig. 5g–i). Also, inhibition of autophagy did not alter viability of *Listeria* (Supplementary Fig. 5j). Overall, SR-BI supported autophagy-dependent elimination of apoptotic cells and dead bacteria but did not alter *Listeria* survival.

### Autophagy activation by SR-BI involves Golgi fragmentation

**Figure 6:**
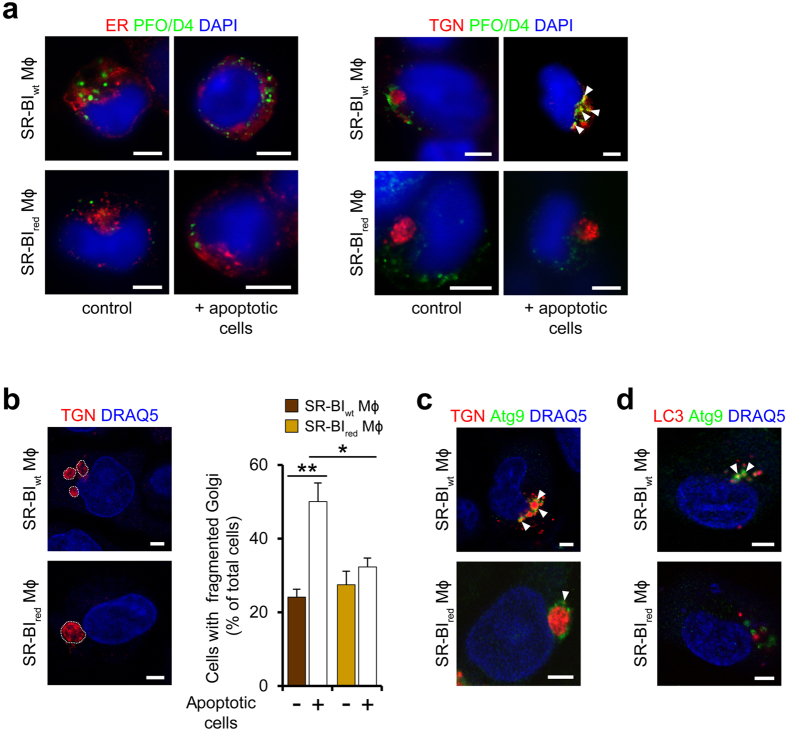
SR-BI promotes release of Atg9-expressing Golgi vesicles. (**a**) Subcellular areas with increased cholesterol levels in SR-BI_wt_ and SR-BI_red_ macrophages exposed to apoptotic neutrophils. Staining with PFO/D4-GFP (green), DAPI (blue, nuclei) and organelle-specific markers erRFP and DsRed-Golgi (both red) for staining of endoplasmic reticulum (ER) and TGN, respectively. Arrowheads indicate colocalization. Confocal microscopy images. Scale bar, 5 μm. (**b**) Scavenger receptor-induced fragmentation of Golgi membranes. Left: Blue (DRAQ5, pseudocolored), nuclei; red, Golgi. Golgi structures delineated with dotted lines. Scale bar, 5 μm. Right: Quantification of Golgi fragments after incubation with apoptotic neutrophils. n = 3 (control) and n = 4 (plus apoptotic cells), 20–30 cells per experiment. *P < 0.05, **P < 0.01. One-way ANOVA with Bonferroni post-test correction. (**c**) Association of Atg9 (green) with Golgi vesicles (red). Colocalization of Atg9 with TGN marker. Arrowheads indicate colocalization. Confocal microscopy images. Scale bar, 5 μm. (**d**) Colocalization of Atg9 (green) with LC3 (red). Confocal microscopy images. Arrowheads indicate colocalization. Scale bar, 5 μm. Data are representative of three different experiments (**a**–**d**).

To specify the subcellular sites of cholesterol accumulation supporting autophagy activation, we visualized areas with high cholesterol levels using PFO/D4, the cholesterol-binding domain of PFO. Both in macrophages exposed to apoptotic cells and in those exposed to vehicle, we found almost no colocalization of such areas with markers specific for endoplasmic reticulum (Fig. 6a) or mitochondria (*not shown*). Instead, in macrophages exposed to apoptotic neutrophils, but not in those incubated with vehicle, the PFO/D4-stained areas co-localized with the trans-Golgi network (TGN) (Fig. 6a). In SR-BI_wt_ macrophages, these areas were associated with Golgi fragments. In contrast, in SR-BI_red_ macrophages the areas were associated with an intact Golgi. Indeed, SR-BI increased the number of Golgi fragments both in human macrophages (Fig. 6b) and CHO cells (Supplementary Fig. 6a).

Since of the major mediators of autophagy only Atg9 resides in TGN membranes and in vesicles shed by them^38^, we analysed its localization in relation to the areas of cholesterol accumulation. Following exposure to apoptotic neutrophils, Atg9 co-localized strongly and almost exclusively with the Golgi fragments in SR-BI_wt_ macrophages while it co-localized less strongly with the Golgi in SR-BI_red_ macrophages (Fig. 6c). Similarly, association of Atg9 with the Golgi fragments in *Listeria*-infected hSR-BI^+^ cells was increased relative to its Golgi association in wt cells (Supplementary Fig. 6b). The Atg9-containing structures were decorated with LC3 in SR-BI_wt_ macrophages but not in SR-BI_red_ macrophages (Fig. 6d). Also, the Atg9-positive structures in hSR-BI^+^ cells co-localized more strongly with LC3 compared to wt cells (Supplementary Fig. 6c).

These results could suggest that SR-BI-induced Golgi fragmentation might participate in the formation of autophagic vesicles. To test this hypothesis, we inhibited Golgi fragmentation with the cPLA2 inhibitor methyl-arachidonyl-fluorophosphonate (MAFP) or the src family kinase inhibitor SU6656^39^,^40^. Both inhibitors prevented autophagy activation in SR-BI_wt_ macrophages but not in SR-BI_red_ macrophages (Supplementary Fig. 6d,e). Notably, MβCD also inhibited Golgi fragmentation, which is in agreement with induction of Golgi fragmentation by local cholesterol accumulation^39^,^40^. The inhibitory effect of MβCD was largely comparable to the one induced by MAFP or SU6656. Hence, scavenger receptor-induced increases in intracellular cholesterol concentrations contributed to activate autophagy by supporting Golgi fragmentation.

### SR-BI promotes autophagy *in vivo* independent of inflammasome activation

**Figure 7:**
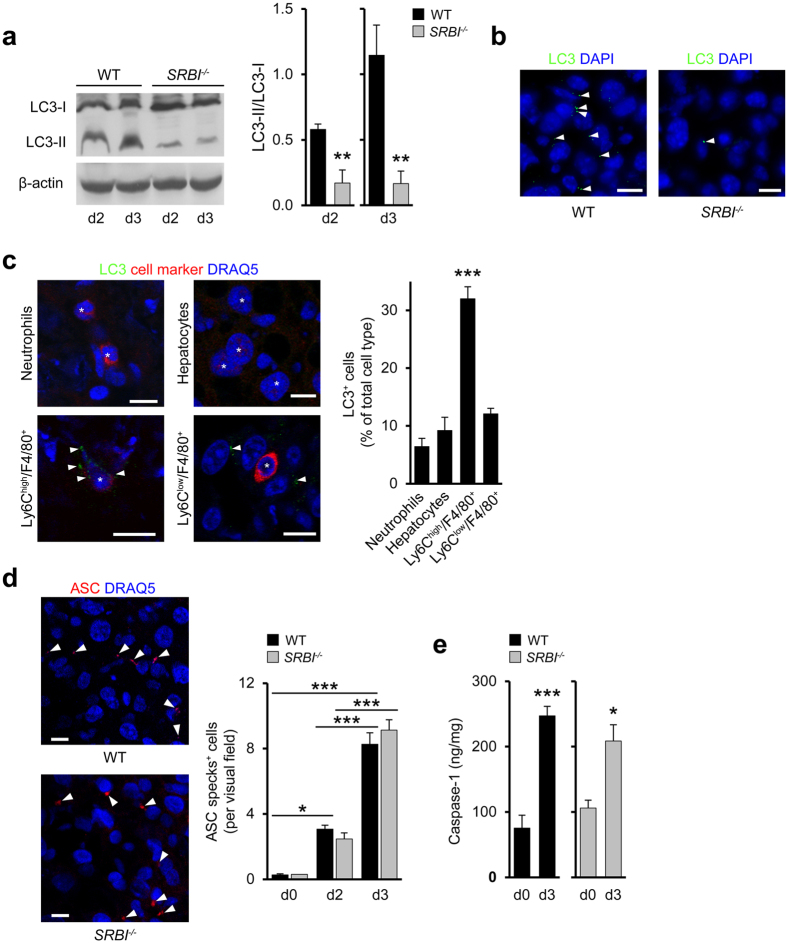
SR-BI induces autophagy without affecting inflammasome activation *in vivo*. (**a**) LC3-I lipidation in the livers of WT and *SRBI*^−/−^ mice during *Listeria* infection. Left: Representative western blots from three different mice. Right: Quantitative analysis of LC3-II formation (expressed as LC3-II to LC3-I ratio). n = 4. (**b**) Confocal microscopy images of LC3 puncta in livers at d3 of *Listeria* infection. Arrowheads indicate LC3 puncta. Scale bar, 20 μm. (**c**) Colocalization of LC3 with neutrophils, hepatocytes, Ly6C^high^ and Ly6C^low^ macrophages in *Listeria*-infected livers of WT mice (d3). Left: Confocal microscopy images. Scale bar, 10 μm. Right: Quantitative analysis of LC3^+^ cells. n = 3, ten images per animal. (**d**) Cells with ASC specks in the livers of *Listeria*-infected mice. Left: Confocal microscopy images: Arrowheads indicate ASC specks (red); Scale bar, 10 μm. Right: Quantitative analyses of ASC specks. n = 2 (d0, *SRBI*^−/−^) and n = 3 (d0, WT; d2, d3, WT, *SRBI*^−/−^), ten images per animal. (**e**) Liver caspase-1 levels in *Listeria*-infected mice. n = 4 (d0), n = 6 (d3). *P < 0.05, **P < 0.01, ***P < 0.001. One-way ANOVA with Bonferroni post-test correction (**c**,**d**) and *t*-test (**a**,**e**). Data are representative of experiments on three different mice (**a**–**d**).

To examine whether SR-BI affected autophagy *in vivo* we determined LC3-II formation in *Listeria*-infected mice. In the livers of WT mice, LC3-I lipidation was markedly increased compared to *SRBI*^−/−^ mice (by 7-fold at d3; Fig. 7a). At d4, LC3-II formation in WT mice was reduced by 36.3 ± 6.1% compared to d3 (n = 3 mice per group), indicating that autophagy induction was transient. These results suggested that SR-BI was largely responsible for activation of autophagy during the peak of *Listeria* infection at d3. In line with this, liver tissue in WT mice but not in *SRBI*^−/−^ mice was highly enriched with LC3 puncta at d3 (Fig. 7b). Furthermore, SR-BI enhanced LC3-II formation and LC3 puncta in *Yersinia*-infected livers (Supplementary Fig. 7a,b). LC3 puncta in *Listeria*-infected WT mice markedly co-localized with Ly6C^high^ macrophages (Fig. 7c), but less so with hepatocytes, Ly6C^low^ macrophages, and neutrophils, indicating that inflammatory macrophages represented major sites of autophagy activation.

Autophagy may regulate activation of different types of inflammasomes^41^, which generate caspase-1 to promote formation of cytokines such as IL-1β. To dissect a potential role of inflammasomes, we visualized oligomers (specks) of the adapter protein apoptosis-associated *speck*-like protein containing CARD (ASC). The number of cells with ASC specks in livers of WT mice markedly increased between d0 and d3 of *Listeria* infection (Fig. 7d 42). Similar increases in ASC specks were seen in *SRBI*^−/−^ mice. Also, liver caspase-1 contents were augmented to a comparable extent during infection in WT and *SRBI*^−/−^ mice (Fig. 7e) and levels of IL-1β were not significantly changed (Supplementary Fig. 7c). Furthermore, *Listeria* infection of human macrophages *in vitro* promoted formation of ASC specks despite large reductions in SR-BI expression (Supplementary Fig. 7d). Hence, SR-BI did not alter activation of inflammasomes.

The scavenger receptors CD36 and SR-AI were robustly expressed in *Listeria*-infected WT and *SR-BI*^−/−^ mice and their expression levels were unaffected by SR-BI deficiency (Supplementary Fig. 7e). Thus, under the conditions employed, CD36 and SR-AI were unable to activate autophagy and could not compensate for the loss of autophagy in *SR-BI*^−/−^ mice. Instead, analyses in *Cav-1*^−/−^ mice showed that Cav-1 increased liver autophagy during *Listeria* infection (Supplementary Fig. 7f). Cav-1 also diminished the number of TUNEL^+^ cells and reduced the area of infectious foci (Supplementary Fig. 7g,h). Bacterial sequestration inside foci was unchanged by Cav-1 (Supplementary Fig. 7i). Liver CFU values were slightly decreased in *Cav-1*^−/−^ mice compared to WT mice (Supplementary Fig. 7j). Thus, Cav-1 promoted autophagy and appeared to protect from organ damage at least in part in a comparable way as SR-BI.

### Autophagy preserves tissue integrity during antimicrobial immune responses

Next, we studied mice with heterozygous deficiency for beclin-1 (mammalian Atg6 homologue), which were previously shown to fail to activate autophagy^43^ (*Becn1*^+/−^). In line with this, both the number of LC3 puncta and the conversion of LC3-I to LC3-II were massively reduced in *Becn1*^+/−^ relative to *Becn1*^+/+^ mice (Supplementary Fig. 8a). Moreover, compared to *Becn1*^+/+^ mice, infection of *Becn1*^+/−^ mice markedly enlarged liver-associated infectious foci (Supplementary Fig. 8b). Increases in foci-associated dead host cells were mostly responsible for this enlargement. Indeed, the percentages of CC3^+^ neutrophils, -Ly6C^high^ macrophages, and -Ly6C^low^ macrophages, but not those of CC3^+^ hepatocytes, were increased in *Becn1*^+/−^ mice relative to *Becn1*^+/+^ mice (Supplementary Fig. 8c).

Moreover, less neutrophils had been engulfed by macrophages in *Becn1*^+/−^ mice compared to *Becn1*^+/+^ mice (Supplementary Fig. 8d), suggesting that clearance of apoptotic corpses was impaired in *Becn1*^+/−^ animals. We also observed increased hepatocyte necrosis in the core of foci of *Becn1*^+/−^ mice relative to *Becn1*^+/+^ mice (Supplementary Fig. 8e). The area of foci strongly correlated with the number of necrotic hepatocytes in *Becn1*^+/+^ and *Becn1*^+/−^ mice (Supplementary Fig. 8f), indicating that the enhanced focus growth was associated with hepatocyte death. Focus area and hepatocyte necrosis were also positively related to each other in *SR-BI*^−/−^ animals (Supplementary Fig. 8f).

**Figure 8:**
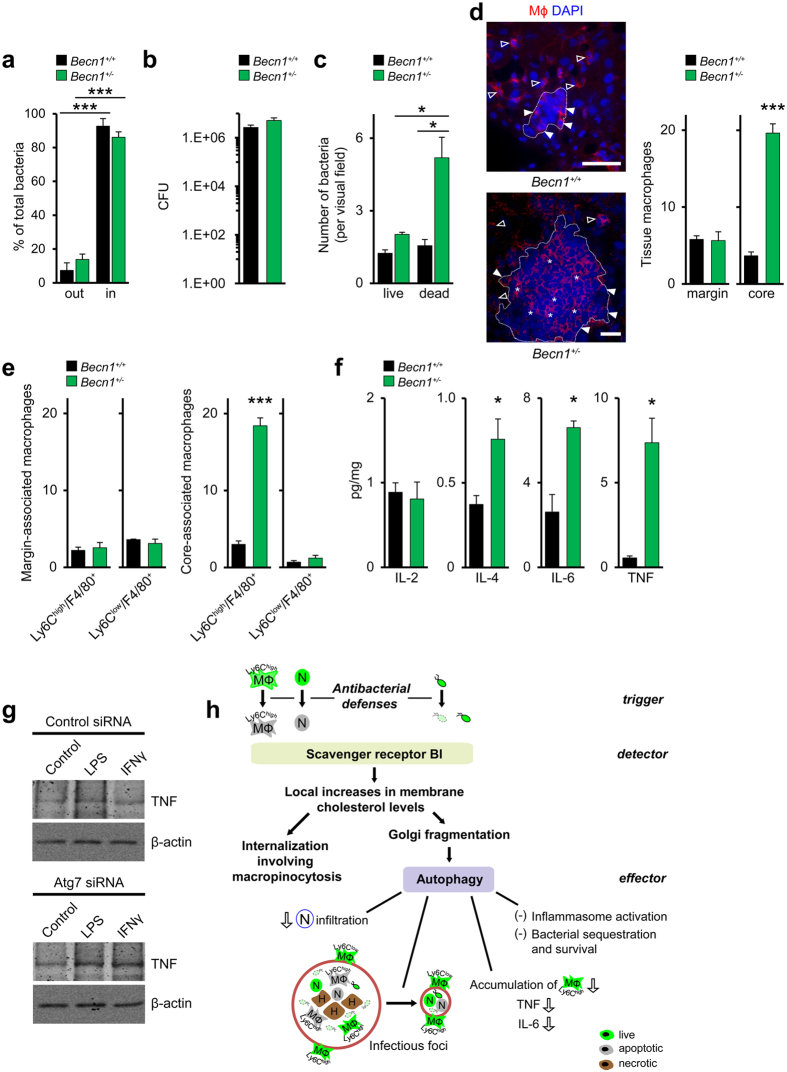
Autophagy prevents tissue deterioration by antimicrobial defenses. (**a**) Distribution of *Listeria* inside and outside of infectious foci. Percentage of total bacteria. n = 3, five images per animal. (**b**) Liver cfu values in *Listeria*-infected *Becn1*^+/+^ and *Becn1*^+/−^ mice (d3). n = 3. (**c**) rRNA-positive and rRNA-negative bacteria in the livers of *Listeria*-infected mice of (d3). n = 3, ten images per animal. (**d**) Macrophage distribution inside and outside of infectious foci (d3). Left: Representative confocal microscopy images. Red, macrophages; blue, nuclei; empty arrowheads, extrafocal macrophages; full arrowheads, macrophages at margins of foci; stars, intrafocal macrophages. Scale bar, 50 μm. Data are representative of experiments on three different mice. Right: Quantitative analysis of macrophage distribution in infectious foci of *Becn1*^+/+^ and *Becn1*^+/−^ mice. n = 3, ten images per animal. (**e**) Ly6C^high^ and Ly6C^low^ macrophages at margin and in core of infectious foci of *Listeria*-infected mice (d3). n = 3, 1 images per animal. (**f**) Tissue cytokine levels in *Listeria*-infected *Becn1*^+/+^ and *Becn1*^+/−^ mice. n = 3 (IL-6, *Becn1*^+/−^), n = 4 (*Becn1*^+/−^), n = 6 (WT). (**g**) Representative western blot (from two different experiments) of TNF formation in LPS- or IFNγ-stimulated SR-BI_wt_ without or with reduced Atg7 expression. (**h**) Model of a bona fide immune pathway selectively controlling immunopathology. SR-BI detects products of antimicrobial defenses such as apoptotic immune cells and dead bacteria as well as (live) bacteria. As a response, SR-BI generates lipid domains which trigger internalization of apoptotic cells and bacteria and activate autophagic responses. *In vivo*, SR-BI- and beclin-1-dependent autophagy restricts neutrophil (N) infiltration, degrades host cell and bacterial corpses and prevents accumulation of Ly6C^high^ macrophages (Mϕ) in organs such as the liver. These mechanisms prevent hepatocyte (H) necrosis and suppress growth of infectious foci. Survival and intrafocal containment of bacteria are unchanged. Thereby, autophagy acts as effector of a surveillance pathway that restricts specifically organ damage by antimicrobial immunity. *P < 0.05, ***P < 0.001. One-way ANOVA with Bonferroni post-test correction (**a**), Kruskal-Wallis One Way Analysis of Variants on Ranks (**c**), *t*-test (**d**–**f**) and Mann-Whitney Rank Sum Test (**f** (TNF)).

Bacterial containment inside foci (Fig. 8a) and organ pathogen burden (Fig. 8b) were unchanged in *Becn1*^+/−^ relative to *Becn1*^+/+^ mice. Nonetheless, foci in *Becn1*^+/−^ animals contained higher numbers of dead bacteria than *Becn1*^+/+^ mice (Fig. 8c). Live bacteria were comparable in the two mouse strains. Furthermore, the number of ASC specks and IL-1β levels in the liver were largely comparable in *Becn1*^+/+^ and *Becn1*^+/−^ mice (Supplementary Fig. 8g,h), indicating that inflammasome activation was unchanged by autophagy deficiency. Tissue levels of live neutrophils and Ly6C^high^ macrophages were augmented in *Becn1*^+/−^ mice compared to control animals (Supplementary Fig. 8i), suggesting that autophagy controlled extravasation of neutrophils and Ly6C^high^ monocytes.

In *Becn1*^+/−^ but not in *Becn1*^+/+^ mice, a large fraction of macrophages resided in the core of foci (Fig. 8d). These intrafocal macrophages in *Becn1*^+/−^ were almost exclusively Ly6C^high^ macrophages (Fig. 8e). Instead, macrophages localized at the margins of foci (perifocal macrophages) represented both Ly6C^high^- and Ly6C^low^ macrophages and their numbers did not differ between *Becn1*^+/−^ and *Becn1*^+/+^ mice. In *SRBI*^−/−^ mice, similar changes in Ly6C^high^ macrophages were seen as in *Becn1*^+/−^ mice (Supplementary Fig. 8j,k). Ly6C^low^ macrophages were lowered at margins of foci and increased in their core. Thus autophagy regulated both the accumulation and tissue localization of Ly6C^high^ macrophages.

Next, we examined whether autophagy affected tissue levels of proinflammatory cytokines which can markedly contribute to tissue damage during infections^5^. Tissue levels of IL-2 and IL-4 were unchanged and elevated, respectively, in *Becn1*^+/−^ mice compared to control mice while the proinflammatory cytokines TNF and IL-6 were increased (Fig. 8f). In *SRBI*^−/−^ mice we observed essentially comparable increases in TNF and IL-6 levels as in *Becn1*^+/−^ mice (Supplementary Fig. 8l). Since macrophages are main sites of autophagy activation (Fig. 7c) we tested whether autophagy regulated macrophage TNF production. Repression of autophagy by inhibition of Atg7 expression enhanced TNF formation in LPS- or IFNγ-stimulated macrophages *in vitro* as compared to control macrophages (Fig. 8g). Hence, autophagy suppressed macrophage release of TNF. Combined, the results showed that the characteristics of organ damage in *Becn1*^+/−^ mice were closely similar to those in *SRBI*^−/−^ mice.

## Discussion

Overall, our findings suggest that protection from immunopathology during acute infection can be supported by a unified defense pathway that appears to be based on a distinct pattern of trigger, detector and effector mechanisms and thus has the characteristics of a bona fide immune process (Fig. 8h). We observed that the assembly of pathogen-controlling inflammasomes^44^, the build-up of microbicidal foci^21^,^45^, as well as the accumulation of bacteria and apoptotic immune cells during acute bacterial infection of the liver and spleen were accompanied by a transient activation of beclin-1-dependent autophagy. Such autophagic responses, the peak of which coincided with the peak of *Listeria* infection, were critically promoted by SR-BI. Autophagy suppressed the tissue infiltration of neutrophils already before the maximum of organ bacterial load was reached. Moreover, it prevented accumulation of live inflammatory macrophages inside infectious foci and directly inhibited the formation of the proinflammatory mediator TNF at the peak of bacterial burden. Together with its ability to promote the clearance of apoptotic immune cells and dead bacteria, this comprehensively protected from the collateral organ damage induced by antimicrobial responses during acute bacterial infection.

At the cell level, protection from damage by antimicrobial immunity was found to be based on a SR-BI-triggered membrane lipid domain/Golgi fragmentation/autophagy axis. The scavenger receptor increased cholesterol levels and formation of membrane lipid domains at distinct subcellular localizations such as in cell membrane ruffles, endosomes and the Golgi. Local accumulation of membrane cholesterol in the cell periphery of macrophages enhanced uptake of live and dead bacteria as well as of apoptotic immune cells via internalization mechanisms sharing characteristics with macropinocytosis (such as inhibition by EIPA) *in vitro*; whether the same internalization mechanisms are also involved in uptake of apoptotic immune cells *in vivo* remains to be determined. In this context it is interesting to note that engulfment and clearance of apoptotic cells via efferocytosis might resemble more macropinocytosis than classical phagocytosis^46^.

The increased formation of cholesterol-enriched lipid domains in Golgi membranes induced by SR-BI was associated with an enhanced fragmentation of this organelle and the release of Atg9-containing vesicles. Such Golgi fragmentation contributed to initiate autophagy which was most likely supported by the ability of the Golgi vesicles to participate in the formation of autophagic vesicles. Future experiments will need to clarify how SR-BI and its ability to regulate membrane cholesterol concentrations exactly control autophagy and whether this involves canonical autophagy or features of LC3-associated phagocytosis^47^. For example, lipid domain formation by SR-BI in intracellular membranes could directly affect lipidation of LC3-I.

The autophagic response activated by SR-BI, which in particular occurred in inflammatory macrophages, did not alter substantially antimicrobial activities. Indeed, containment of bacteria inside foci, bacterial survival as well as the formation of ASC specks common to all types of inflammasomes were not affected in *Becn1*^+/−^ mice that fail to induce autophagy. Nonetheless, autophagy suppressed the tissue accumulation of live inflammatory macrophages and extravasation of neutrophils and directly inhibited the formation of the proinflammatory mediator TNF. Together with its ability to promote the clearance of apoptotic immune cells and dead bacteria, this comprehensively protected from the collateral organ damage induced by antimicrobial responses at the peak of bacterial burden. Such protection from immunopathology during early stages of infection is likely particularly effective since it reduces the requirement for recruitment and/or activation of potentially deleterious immune cells as well as the need for elaborate repair and regeneration mechanisms.

Several of the molecular and cellular mediators previously revealed to prevent immunopathology under infectious and sterile conditions can be protective under certain conditions but harmful in other contexts. For example, type I IFN can reduce immunopathology in chronic viral infections but supports host damage during acute viral infections^48^. In line with this, several of the molecules and cellular pathways identified here to suppress organ immunopathology during acute bacterial infection can support vastly differing functions since for example regulation of proinflammatory cytokine formation by autophagy is markedly context-dependent^49^. Thus, together with previous studies our work suggests that the specificity of protection from tissue immunopathology might originate in particular from the distinct interplay of the individual molecular and cellular components involved.

Another determinant of the specificity of the defense pathway described here is the differential outcome of the autophagic response to dead bacteria versus live bacteria. The inability of SR-BI-induced autophagy to eliminate *Listeria* and *Yersinia* is in line with previous studies indicating that autophagy often fails to kill bacteria *in vivo*^50^. This could be due to the ability of pathogens, including *Listeria* and *Yersinia*^51^,^52^, to escape from this ancient defense mechanism. Nonetheless, provided that *Listeria* participate in autophagy activation in a similar way *in vivo* as observed here *in vitro*, live bacteria could indirectly help to protect against immunity-induced tissue damage. The lack of an inhibitory effect of SR-BI-triggered autophagy on inflammasome activation appears to be at variance with previous work indicating that autophagy suppresses activation of inflammasomes^53^. However, autophagy can also positively regulate NLRP3-mediated IL-1β secretion^54^ and the interaction between autophagy and inflammasomes is in general context-dependent^55^.

Infectious foci or granuloma are as confirmed here major sites of pathogen containment^56^ and elimination^21^,^45^ but can also be triggers of tissue damage. Indeed their dysregulated growth as induced in particular by accumulation of inflammatory macrophages and neutrophils in their core region resulted in tissue necrosis both in *Becn1*^+/−^ and *SRBI*^−/−^ mice. Overall, our findings suggest a coupling between protection from immunopathology by antibacterial defenses, host lipid homeostasis and control of infectious foci which could potentially be of relevance for the understanding of the regulation of atherosclerotic plaque development. Indeed, infectious foci and atherosclerotic plaques exhibit several structural similarities^57^ that include among others the presence of a core region containing apoptotic and necrotic cells as well as live macrophages accumulating at the margins of these structures. In line with this, SR-BI in addition to suppressing growth of the core of infectious foci as documented here also inhibits plaque development in particular by restricting growth of the plaque core^58^.

## Materials and Methods

### Bacterial infections

Homozygous *SRBI*^−/−^ mice^59^ (also deficient for SR-BII, the splice variant of SR-BI; donated in part by Deneys van der Westhuyzen and Miranda van Eck), *Becn1*^+/−^ mice^43^ (Jackson Laboratories), *Cav-1*^−/−^ mice (donated by Teymuras Kurzchalia) or the respective WT mice (C57BL/6) were inoculated intraperitoneally with either *Listeria monocytogenes* (strain EGD) or *Yersinia enterocolitica* O:3 (strain 108-P, 5 × 10^6 ^cfu in 100 μl PBS). In general, after 2 and 3 days (*Listeria*) or 4 days (*Yersinia*), the mice were killed to harvest liver, spleen and kidney. In parallel, blood was drawn from the left ventricle of the heart. The number of viable bacteria (cfu) in the homogenates of the liver and spleen were analysed. The studies were carried out in accordance with the approved guidelines of the local committee of laboratory animal care (Regierung von Oberbayern, Munich, Germany) and German Laws on animal welfare (Tierschutzgesetz). All experimental methods were approved by these institutions.

### Mammalian cells

For isolation of mouse macrophages, mice received an intraperitoneal injection of thioglycollate (4%). Cells were collected after 4 days by peritoneal lavage, centrifuged, resuspended in RPMI medium (10% FBS) and plated onto μ-dishes (Ibidi) for 24 h.

Human THP-1 monocytes were stimulated with PMA (300 nM, Merck) overnight to differentiate them into macrophages, which were then transfected with SR-BI-specific siRNA oligonucleotides or control oligonucleotides (Qiagen) for 48–72 h. Human neutrophils were isolated from whole blood using a density gradient separation method with dextran (3%, MW 500,000, Roth) containing HBSS/Hepes (10 mM) medium. After centrifugation, separation of the neutrophil layer and lysis of residual erythrocytes, the neutrophils were washed and resuspended in RPMI/Hepes (10 mM). Neutrophil apoptosis was induced by overnight incubation with pyocyanin (25 μM, Sigma). The apoptotic neutrophils were stained with TAMRA (50 μM, Thermo Fisher Scientific) and incubated for 30 min with macrophages at 37 °C.

CHO cells were stably transfected with the cDNA for human SR-BI^14^. Transient transfections with different plasmids for murine SR-BI (native mSR-BI and its mutants SRCDSR and CDSRCD^27^, donated by Margery Connelly), were performed using Lipofectamine^®^2000 (Thermo Fisher Scientific).

### Cell entry of bacteria and engulfment of apoptotic cells

Isolated WT, *SRBI*^−/−^ and *Cav-1*^−/−^ macrophages as well as SR-BI_wt_ and SR-BI_red_ macrophages were incubated for 30–45 min with *Listeria* (MOI 10), apoptotic neutrophils (10:1) or dead *Listeria* (60 min treatment of *Listeria* at 70 °C) (50:1). To assess the participation of pinocytic internalization pathways, cells were incubated with 70 kDa fluorescein-labeled dextran (0.5 mg/ml, Thermo Fisher Scientific) for different time periods. Macropinocytosis was inhibited by preincubation with different concentrations of EIPA (Sigma). To deplete membrane cholesterol, cells were preincubated for 30–45 min with MβCD (5 mM) in media without FBS. In all experiments analyzing the effect of cholesterol depletion on autophagy, MβCD was added after the end of the internalization period of bacteria or apoptotic cells.

For visualization of co-internalization of *Listeria*, dead bacteria or apoptotic cells with dextran, bacteria (labeled with FM 4-64FX) or apoptotic cells labeled with TAMRA were added to the macrophages together with 70 kDa fluorescein-labeled dextran. The cells were fixed and immediately visualized by confocal microscopy (cLSM510, Zeiss). In other experiments, the cells were washed and the plasma membrane was stained using FM 4-64FX (10 μM). The cells were fixed and visualized by confocal microscopy. To quantify the fluorescence intensity from the intracellular bacteria or apoptotic cells, the plasma membrane staining was used to set regions of interest (ROI) and the fluorescence intensity was calculated using ZEN software (Zeiss).

### LC3 puncta

The plasma membrane of cells was stained with FM 4-64FX. After 3 washing steps, cells were fixed and permeabilized. Cells were immunostained using anti-LC-3 antibody (10 μg/ml, Novus) and detected with Alexa488-labeled secondary antibody. Cells were visualized by confocal microscopy.

### ASC specks *in vitro* and *in vivo*

To identify ASC specks, cells were stained with ATTO647N-labeled anti-ASC antibody (5 μg/ml, Adipogen) and either plasma membrane marker FM 4-64FX (*in vitro*, Thermo Fisher Scientific) or nuclear marker DRAQ5™ (*in vivo*, Abcam).

### Determination of membrane order

Cells labeled with Laurdan (10 μM, Molecular Probes) were visualized with Leica TCS SP5 confocal microscope. Submembraneous areas were defined as areas extending 1 μm towards the cell interior. Laurdan was excited at 800 nm with a mode-locked titanium sapphire laser (MaiTai HP laser, Spectra-Physics). An amplitude modulator linked to the Leica Microsystems software system controlled two-photon intensity input. To record intensities at selective wavelengths, internal photon multiplier tubes collected images as 8-bit, unsigned images with 512 × 512 pixels at 400 Hz scan speed. Laurdan image calculations were carried out in ImageJ (NIH). The generalized polarization (GP) was defined as


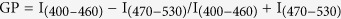


and calculated for each pixel using the two Laurdan intensity images.

### Western blots

For SR-B1, LDLR, LRP1, SR-A1, and CD36 expression analyses, proteins of liver homogenates (50 μg total protein/lane) were separated by SDS-PAGE (8%) and transferred onto polyvinylidene difluoride membranes. The membranes were blocked with BSA (5%) and incubated with anti-SR-BI (1:1000, Novus), anti-LRP1 (1:500, SantaCruz), anti-LDLR (1:500, SantaCruz), anti-SR-AI (1:500, SantaCruz) and anti-CD36 antibodies (1:500, SantaCruz). This was followed by incubation with horseradish peroxidase (HRP) linked secondary IgGs (1:3000, SantaCruz) and visualization of the proteins by enhanced chemiluminescence (PerkinElmer).

To determine TNF formation by macrophages, THP-1 monocytes were stimulated with PMA to differentiate them into macrophages and then transfected with either control or Atg7-specific siRNA for 48 h. Transfected macrophages were then treated with either vehicle, LPS (100 ng/ml) or IFNγ (20 ng/ml) for 18 h. Macrophages were collected and lysed with Triton X-100 (0.4%). Subsequently, the lysate was centrifuged at 15,000 × g for 15 min; the supernatant was removed and kept on ice. Lysates (50 μg/lane) were separated by SDS-PAGE (10%) and transferred onto polyvinylidene difluoride membranes. After incubation with anti-TNFα antibody (1:1000, Genzyme), membranes were incubated overnight with HRP linked secondary IgG and the proteins were visualized by enhanced chemiluminescence.

### Intracellular degradation of apoptotic cells and dead bacteria

We incubated macrophages for 30 min with apoptotic neutrophils (TAMRA-stained) or dead *Listeria* (SYTOX-green-stained, 3 μM, Thermo Fisher Scientific) to induce their engulfment. To inhibit autophagy, human macrophages were transfected with Atg7-specific siRNA oligonucleotides or control oligonucleotides (Qiagen) for 48 h. In other cases, the cells were treated after the internalization period with DMSO (control), wortmannin (100 nM) or 3-MA (10 mM) to inhibit autophagy. Degradation of engulfed apoptotic cells and internalized dead bacteria was determined by the fluorescence intensity of intracellular TAMRA or SYTOX-green signals by confocal microscopy using the FM 4-64FX-labeled plasma membrane to define ROIs.

### Golgi fragmentation

To visualize Golgi fragmentation, human macrophages and CHO cells were transfected with DsRed-galT1 or CFP-galT1 plasmid (donated by M. Lorenz, Munich, Germany) using Torpedo^DNA^ (Ibidi). After 24 h, macrophages were incubated with apoptotic neutrophils for 30 min. CHO cells were infected for 45 min at 37 °C with *Listeria*. Either the nuclei (DRAQ5™, 5μM, Thermo Fisher Scientific) or the plasma membrane (using FM 4-64FX) were stained. Cells were fixed (4% PFA) and analyzed by confocal microscopy. Cells that contained >3 clearly separated DsRed- or CFP-labeled structures over the entire cell body were classified as being positive for Golgi fragments.

To visualize the co-association of the Golgi with Atg9, human macrophages and CHO cells were co-transfected with DsRed-galT1 to label the Golgi compartment and Atg9-EGFP (provided by Y. Takahashi and S.W. Scherer, Toronto, Canada). After the transfections, the cells were incubated with apoptotic neutrophils or in case of CHO cells infected with *Listeria*.

In other experiments, the Atg9-EGFP transfected cells were incubated with apoptotic neutrophils or infected with *Listeria* as described above, and stained with anti-LC3 antibody (10 μg/ml; Novus) for 2 h, followed by addition of Alexa594-labeled secondary antibody (1:250; Thermo Fisher Scientific). Cells were analyzed by confocal microscopy. To inhibit Golgi vesiculation, human macrophages and CHO cells were preincubated with DMSO (control; Roth), MAFP (50 μM, 30 min; Merck), or SU6656 (25 μM, 12 h; Calbiochem) followed by incubation with apoptotic neutrophils or bacteria.

### Association of membrane areas with increased cholesterol levels with intracellular organelles

To label endoplasmic reticulum, TGN, and mitochondria, human macrophages and CHO cells were transfected with the plasmids PR-CD5L-HA-erRFP (provided by F.X. Pimentel-Muíños, Salamanca, Spain), DsRed-galT1 (donated by M. Lorenz, Munich, Germany), and pOct-YFP (provided by K. Hell, Munich, Germany), respectively. All transfections were performed with Torpedo^DNA^ (Ibidi). After the transfection, cells were incubated for 30 min at 37 °C with apoptotic neutrophils or were infected for 45 min at 37 °C with *Listeria* in the presence of PFO/D4-GFP to visualize the association of membrane areas of increased cholesterol levels with the organelles. Subsequently, the cells were fixed, mounted in Vectashield^®^ with DAPI and analyzed by confocal microscopy.

### Immunohistochemistry

Cryo sections (10 μm) were prepared from mouse tissues. After fixation in methanol/acetic acid for 90 s at −20 °C, sections were washed immediately in PBS. The sections were permeabilized/blocked with saponin solution, incubated with anti-*Listeria* antibody (10 μg/ml, Acris), washed, and stained with Alexa488-labeled secondary antibody (2 μg/ml, Thermo Fisher Scientific). Sections were washed and mounted in Vectashield^®^ with DAPI (Vector Laboratories) for visualization by confocal microscopy.

To determine the amount of tissue-associated neutrophils and macrophages, liver sections were fixed and blocked as described above. The sections were incubated with anti-Ly6G antibody (neutrophils, 1:500, BD Pharmingen) or anti-F4/80 antibody (macrophages, 10 μg/ml, Abcam), followed by staining with Alexa594-labeled secondary antibody (2 μg/ml, Thermo Fisher Scientific). To visualize the internalized neutrophils in macrophages, the slides were co-stained with the cell-specific antibodies.

For detection of different macrophage subsets, tissue macrophages were stained with anti-F4/80 antibody, detected by an Alexa594-labeled secondary antibody. To specifically stain inflammatory macrophages an Alexa555 conjugated anti-Ly6C antibody (2 μg/ml, Abcam) was used. The sections were mounted in Vectashield^®^ with DAPI (Vector Laboratories).

For LC3 staining, sections were incubated with anti-LC3 antibody (10 μg/ml, Novus), followed by staining with Alexa488-labeled secondary antibody (2 μg/ml, Thermo Fisher Scientific). To examine the colocalization of LC3 with different cell types, we incubated the sections with antibodies for neutrophils (anti-Ly6G antibody, 1:500, BD Pharmingen) and hepatocytes (anti-HNF4α antibody, 5 μg/ml, Abcam), followed by incubation with Alexa550-labeled secondary antibody (2 μg/ml, Thermo Fisher Scientific). The nuclei were stained with DRAQ5™.

For detection of tissue-associated apoptotic cells, the sections were labeled with anti-CC3 antibody (1:200, Cell Signaling), followed by incubation with an Alexa488-labeled secondary antibody (2 μg/ml, Thermo Fisher Scientific). For colocalization analysis with cell specific markers for neutrophils, hepatocytes and the different subpopulations of macrophages, the staining was done as described above.

To detect apoptotic plus necroptotic cells in tissue, the TdT-mediated dUTP Nick End Labeling (TUNEL) assay was performed using the ApopTag Fluorescein *In Situ* Apoptosis Detection Kit (Millipore). Nuclei were stained with DAPI or DRAQ5™. TUNEL^+^ structures with a diameter of <4 μm localized in extranuclear compartments were classified as apoptotic bodies.

The total number of dead host cells was estimated by counting the number of TUNEL^+^ cells and/or of cells with fragmented or pyknotic nuclei (nuclear diameter of <30% of average diameter of given cell type as verified using z-stacks). Cells lacking signs of cell death were identified as TUNEL^−^ cells with an intact and normal-sized nucleus (range of 70–130% of average size) and by cell-specific staining as describe above. To detect necrotic hepatocytes, tissue sections were fixed and stained with the Dako EnVision^+^ System-HRP (DAB) kit (Dako) in combination with anti-HNF4α antibody. According to the company’s instructions, the tissue sections were counterstained with hematoxylin/eosin and visualized with a fluorescence microscope (Axio Imager, Zeiss).

### Detection of live and dead *Listeria*

Tissue sections from WT and *SRBI*^−/−^ animals were fixed in methanol:acetic acid and washed with PBS. The sections were subjected to partial lysis using lysis solution for *in situ*-detection of *Listeria* rRNA (Miacom) and then kept for 5 min at 52 °C. Sections were immersed in ethanol (5 min), after the alcohol was evaporated, Atto550-labeled oligonucleotides complementary to *Listeria* rRNA (*Listeria* beacon, Miacom) were applied. After hybridization (15 min, 52 °C), the reaction was terminated by stop solution (Miacom). Sections were dipped in ethanol for 1 min and blocked for 1 h using BSA (5%). After washing, sections were incubated with anti-*Listeria* antibody (20 μg/ml, GeneTex), followed by incubation with Alexa488-labeled secondary antibody. The sections were mounted in Vectashield^®^ with DAPI. Bacteria positive for both anti-*Listeria* antibody and *Listeria* rRNA were classified as live bacteria while bacteria positive for anti-*Listeria* antibody but negative for *Listeria* rRNA were classified as dead bacteria. To verify the specificity of detection of live and dead bacteria, control experiments were performed using live bacteria and dead bacteria suspensions (prepared by heat-killing, 70 °C, 1 h) incubated with tissue sections for 15 min at 37 °C. Sections were fixed, treated exactly as described above and subsequently analyzed using the labeled anti-*Listeria* antibody and *Listeria* beacon. Thereby 97% of the bacteria were positive for both anti-*Listeria* antibody and *Listeria* rRNA (after incubation with live bacteria) and 98% were only positive for anti-*Listeria* antibody (incubation with dead bacteria).

### Cholesterol contents

Cholesterol was extracted from tissues and cells with chloroform:isopropanol:NP-40 (7:11:0.1) or with isopropanol, respectively. Cholesterol contents were quantified by kits (MBL or Wako) or gas liquid chromatography.

### Infectious foci

Clusters of bacteria and host cells (>6 cells) were defined as infectious foci. The foci were visualized in histological tissue preparations by labeling of nuclei with DAPI or DRAQ5™ or by staining with hematoxylin/eosin.

### Caspase-1, cytokine and ALT levels

Caspase-1 levels were measured using an ELISA kit that detects the proform as well as the p10 and p20 domains of mouse caspase-1 (Adipogen). Cytokine contents of liver tissue were determined by ELISA (Quansys Biosciences). Plasma levels of ALT, a measure of hepatocyte necrosis, were determined by photometric measurement of NADH consumption.

### Statistics

All mean values are given ± s.e.m. The results were compared by One way ANOVA for multiple comparisons, Kruskal-Wallis One Way Analysis of Variants on Ranks, Mann-Whitney rank-sum test or two-tailed unpaired *t*-test. P values <0.05 were considered to be significant. *P < 0.05, **P < 0.01, ***P < 0.001.

## Additional Information

**How to cite this article**: Pfeiler, S. *et al*. Distinct surveillance pathway for immunopathology during acute infection via autophagy and SR-BI. *Sci. Rep*. **6**, 34440; doi: 10.1038/srep34440 (2016).

## Supplementary Material

Supplementary Information
