# Supplementary material for: Distinct surveillance pathway for immunopathology during acute infection via autophagy and SR-BI
Source: Sci Rep. 2016 Oct 3;6:34440. doi: 10.1038/srep34440 (PMC5046072; doi:10.1038/srep34440)
Supplement: Supplementary Information [file srep34440-s1.pdf]

Supplementary Information

Distinct surveillance pathway for immunopathology during acute infection via autophagy and SR-BI

Susanne Pfeiler, Avinash B. Khandagale, Astrid Magenau, Maryana Nichols, Harry F.G. Heijnen, Franz Rinninger, Tilman Ziegler, Stephanie Seveau, Sören Schubert, Stefan Zahler, Admar Verschoor, Eicke Latz, Steffen Massberg, Katharina Gaus, Bernd Engelmann

Corresponding author: Bernd.Engelmann@med.uni-muenchen.de

Table of Contents:

Supplementary Methods

Supplementary Figures

Supplementary Methods

Hepatocyte isolation

To isolate mouse hepatocytes, the hepatic artery was perfused with EBSS (without Ca^{2+}/Mg^{2+}), followed by infusion of EBSS (with Ca^{2+}/Mg^{2+}) containing collagenase (0.5 mg/ml) and aprotinin (0.1 µg/ml). The liver was removed, the capsule was cut with a scalpel and the separated cells were resuspended in EBSS (without Ca^{2+}/Mg^{2+}) and centrifuged. The pelleted cells were examined for viability and cell specificity and seeded onto µ-dishes (Ibidi) in Williams'medium E (Thermo Fisher Scientific, 10% FCS).

Bacterial survival *in vitro*

Cells were incubated with *Listeria* (MOI 10) in different media and then incubated with media containing gentamicin (10 µg/ml, 15 - 60 min) in the presence or, in some cases, absence (MβCD) of FBS (10%). In other cases, macrophages were treated after the internalization period (30 min) with DMSO (control), wortmannin (100 nM) or 3-MA (10 mM) to inhibit autophagy. The time intervals given refer to the total time period from the start of coincubation of cells with bacteria until the end of incubation with gentamicin. Following incubation with gentamicin, the cells were washed, lysed with Triton X-100 (0.2%) and cfus were measured in the cell lysates. Control experiments with labeled *Listeria* indicated that after the incubation with gentamicin no bacteria adhered to the extracellular surface of the cell membrane.

Detection of membrane areas with increased cholesterol contents

Localization of cholesterol-rich domains was analyzed using perfringolysin O (theta toxin,BCΘ, provided by Yoshiko Ohno-Iwashita, Iwaki, Japan). The toxin was digested with subtilisin Carlsberg and biotinylated (Waheed, A. A. *et al.* 2001). To determine local cholesterol accumulation in the plasma membrane, wt and hSR-BI+ cells were incubated for

45 min with BCΘ (15 µg/ml, 4°C). BCΘ and SR-BI were visualized under confocal microscope using PE-labeled anti-biotin antibody (BioLegend) and anti-SR-BI antibody (1:200, Novus) detected with Alexa488-labeled anti-rabbit IgG. For labeling of intracellular compartments, the cells were incubated for another 10 min at 37°C. To assess colocalization of the domains with SR-BI in intracellular compartments, cells were permeabilized using saponin. In other experiments, we used the perfringolysin-O-domain 4-GFP construct (PFO/D4-GFP, donated by B. Mutus, Windsor, Canada) to identify cholesterol-enriched membrane domains. Cells were incubated for 45 min PFO/D4-GFP (20 µg/ml, 4°C). For labeling of intracellular compartments, the cells were first incubated for 45 min with PFO/D4-GFP (20 µg/ml, 4°C). Unbound toxin was removed by washing and the cells were incubated for another 10 min at room temperature. The plasma membrane was labeled with FM 4-64FX. To detect lipid bodies, after a 30 min preincubation (with or without MβCD), macrophages were stained with BODIPY493/503 (10 µg/ml, 30 min, Thermo Fisher Scientific) and in parallel with FM 4-64FX. To prevent formation of lipid bodies, cells were preincubated with C75 (24 h, Sigma), an inhibitor of fatty acid synthase (FAS).

Immunoelectron microscopy

We fixed wt and hSR-BI+ CHO cells in PFA (2%) and glutaraldehyde (0.2%) (in 0.1 M phosphate buffer (pH 7.2)). The cells were infiltrated in sucrose (2.3 M) and frozen in liquid nitrogen. 50 - 60 nm-thick sections were cut at -120°C using an Ultracut S (Leica) equipped with an antistatic device (Diatome) and a diamond knife (Drukker). Sections were collected on formvar-coated grids using methylcellulose (1.8%) and sucrose (2.3 M). Immunogold double labeling experiments were conducted on thin frozen sections using 10 and 15 nm gold particles. After labeling, sections were fixed with glutaraldehyde (1%), counterstained for 5 min with uranyl-oxalate (pH 7.0), washed, and embedded in a mixture of methylcellulose (1.8%) and uranyl acetate (0.3%, 4°C). The sections were viewed with a JEOL 1200EX

electron microscope. Localization of cholesterol-enriched membrane domains was performed using biotinylated BCΘ. For detection of plasma membrane cholesterol, cells were incubated with BCΘ for 45 min (15 µg/ml, 4°C). To visualize intracellular cholesterol accumulation, we incubated the cells for another 10 min (37°C). BCΘ was detected on ultra-thin cryosections using anti-biotin antibody (Sanver Tech) and protein A-gold 10 nm. SR-BI was viewed with anti-SR-BI antibody (Abcam) and protein A-gold 15 nm.

Immunohistochemistry

For filipin staining, we thawed freeze-dried tissue sections and rehydrated them in PBS for 30 min. The sections were incubated with primary anti-pan cadherin antibody (1:100, Abcam) to selectively stain plasma membranes and were then treated with Alexa543-labeled secondary antibody (1:400, Thermo Fisher Scientific) and filipin (25 µg/ml, Sigma). Tissue samples were mounted with glycerol and observed by confocal microscopy.

Supplementary Figures

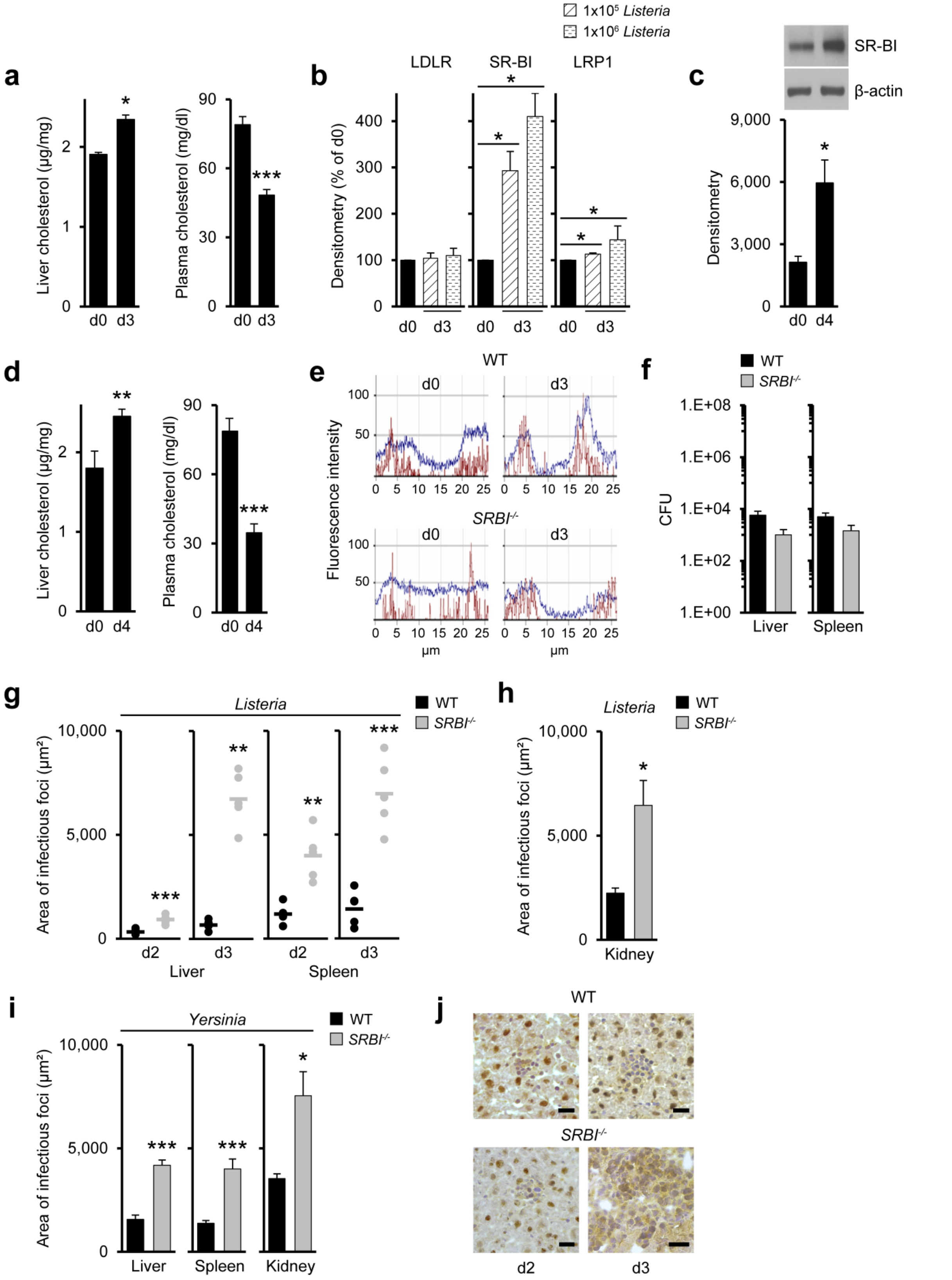

Supplementary Fig.1| Tissue cholesterol distribution and protection from organ damage during bacterial infection. (**a**) Liver (left) and plasma (right) cholesterol contents in *Listeria*-

infected mice. d0, before infection. n = 3 (d0, liver), n = 11 (d3, liver), n = 6 (d0, plasma) and n = 12 (d3, plasma). (**b**) Expression of liver cholesterol transporters in *Listeria*-infected mice. Densitometric quantification. Superscripts refer to pathogen loads. n = 3. (**c**) Liver SR-BI expression in *Yersinia*-infected WT mice (d4). Representative western blots from three different mice (top) and densitometric quantification (bottom). n = 3. (**d**) Liver (left) and plasma (right) cholesterol contents in *Yersinia*-infected mice. n = 3 (d0), n = 8 (d4). (**e**) Cholesterol localization in liver cells before and after *Listeria* infection *in vivo*. Staining for cholesterol (blue, filipin) and plasma membrane (red, cadherin). Representative histograms show fluorescence intensity distribution across single hepatocytes. (**f**) Bacterial survival in *Yersinia*-infected liver and spleen of WT and *SRBI*$^{-/-}$ mice (d4). n = 8 (WT), n = 7 (*SRBI*$^{-/-}$). (**g**) Area of infectious foci in *Listeria*-infected organs. n = 5 (liver, d3; spleen, d2 and d3) and n = 6 (liver, d2), five to ten images per animal. (**h**) Area of infectious foci in kidneys of *Listeria*-infected mice (d3). n = 3, five images per animal. (**i**) Area of infectious foci in *Yersinia*-infected organs of WT and *SRBI*$^{-/-}$ mice (d4). n = 8 (WT, liver, spleen), n = 7 (*SRBI*$^{-/-}$, liver, spleen) and n = 4 (kidney), five images per animal. (**j**) Necrotic HNF4α^{+} hepatocytes in infectious foci. Fluorescence microscopy images. Scale bar, 20 µm. *P < 0.05, **P < 0.01, ***P < 0.001. Kruskal-Wallis One Way Analysis of Variance on Ranks (**b**), *t*-test (**a**, **c**, **d**, **g**, **h**, **i**), Mann-Whitney Rank Sum Test (**g** (liver, d3), **i** (spleen). Data are representative of experiments on three different mice (**e**, **j**).

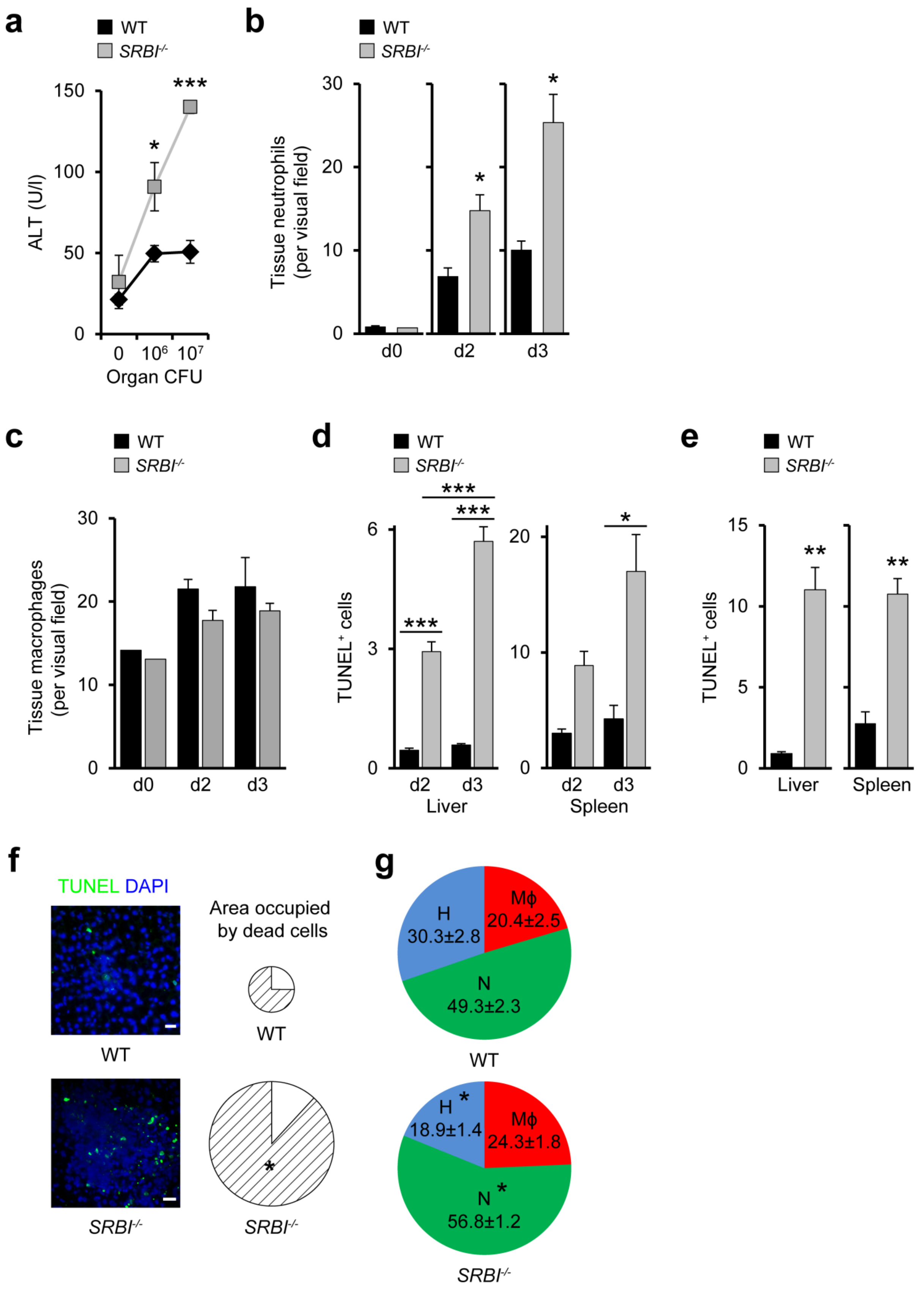

Supplementary Fig. 2| Effect of SR-BI on organ injury indicators during bacterial infection. (**a**) ALT values as a function of liver pathogen burden during *Listeria* infection. n =

3 (*SRBI*$^{-/-}$, control and 10^7), n = 6 (WT, control; *SRBI*$^{-/-}$, 10^6), n = 7 (WT, 10^6) and n = 10 (WT, 10^7). (**b**) Tissue neutrophils in *Listeria*-infected mice. Values refer to total number of liver-associated Ly6G$^+$ cells. n = 2 (*SRBI*$^{-/-}$, d0) and n = 3 (WT, *SRBI*$^{-/-}$), ten images per animal. (**c**) Time-dependent changes in tissue macrophages in *Listeria*-infected mice. Values refer to total number of F4/80$^+$ cells. n = 3 (d2, d3) and n = 2 (d0), ten images per animal. (**d**) Number of TUNEL$^+$ cells per visual field in *Listeria*-infected mouse organs at indicated days of infection. n = 3 (d3, liver), n = 6 (d2, liver and spleen) and n = 5 (d3, spleen), ten images per animal. (**e**) Number of TUNEL$^+$ cells per visual field in *Yersinia*-infected mouse organs (d4). n = 3, ten images per animal. (**f**) Accumulation of dead cells (TUNEL$^+$ cells and/or cells with pyknotic nuclei) in infectious foci of *Listeria*-infected WT and *SRBI*$^{-/-}$ mice (d3). Left: Confocal microscopy images. Scale bar, 20 µm. Data are representative of experiments on three different mice. Right: Quantitative analysis of focus area occupied by dead cells in *Listeria*-infected WT and *SRBI*$^{-/-}$ mice. Shaded area shows area occupied by dead cells as percentage of total focus size. Significance symbol refers to the difference between the percentage of shaded area in WT versus *SRBI*$^{-/-}$ mice. n = 3, five images per animal. (**g**) TUNEL$^+$ cell types in *Listeria*-infected livers (d3). Percentages of hepatocytes (H), macrophages (Mϕ) and neutrophils (N) positive for TUNEL staining. > 90% of TUNEL$^+$ cells were localized in infectious foci. n = 3, seven images per animal. Sum of three cell types set as 100%. Significance symbol refers to the difference between WT versus *SRBI*$^{-/-}$ mice. *$P < 0.05$, **$P < 0.01$, ***$P < 0.001$. One-way ANOVA with a Bonferroni post-test correction (**a**, **d** (liver)), Kruskal-Wallis One Way Analysis of Variants on Ranks (**d** (spleen)), *t*-test (**b**, **e**, **f**, **g**).

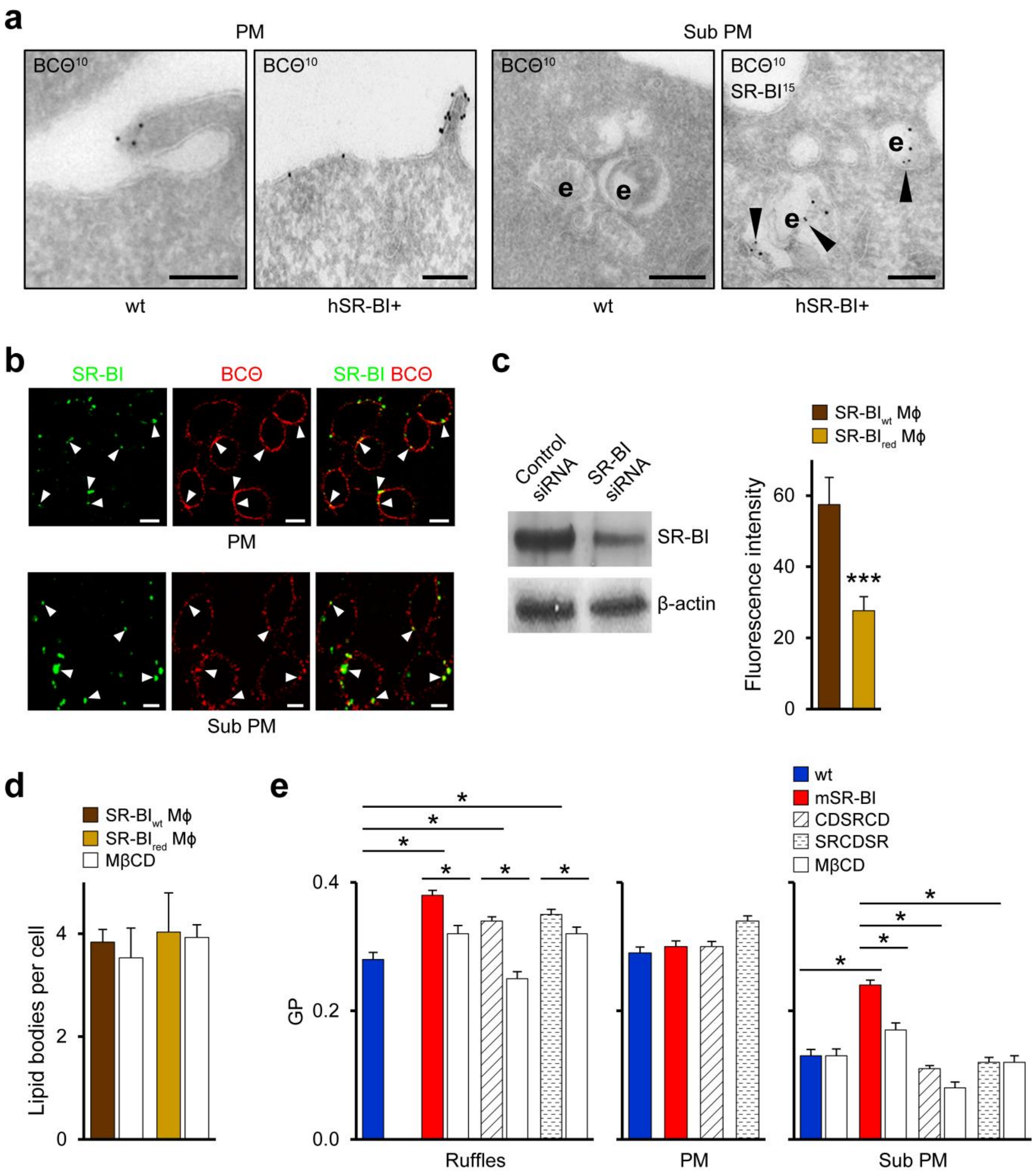

Supplementary Fig. 3| Subcellular localizations of increases in membrane cholesterol levels mediated by SR-BI. (**a**) Visualization of cholesterol-enriched membrane domains with BCΘ. PM, plasma membrane; Sub PM, submembraneous area; wt, wt CHO cells; hSR-BI+, CHO cells expressing hSR-BI; e, early endosomes. 10 and 15 nm indicate size of gold particles. Arrowheads indicate colocalization of both types of gold particles. Immunoelectron microscopy. Scale bars, 200 nm. (**b**) Colocalization of SR-BI with membrane areas of local cholesterol accumulation. Confocal microscopy images show BCΘ-labeled cholesterol-

enriched membrane domains (red) that associate with SR-BI (green) in hSR-BI+ cells (arrowheads). Scale bars, 10 µm. (**c**) SiRNA-mediated reduction of SR-BI expression in THP-1 macrophages (SR-BI_{red}; left). Right: Membrane areas with increased cholesterol levels in macrophages. Quantification of cholesterol-enriched membrane domains in plasma membrane by staining with PFO/D4-GFP in THP-1 macrophages with normal SR-BI expression (SR-BI_{wt}) and SR-BI_{red} macrophages. 3 independent experiments with ten images per experiment. (**d**) Influence of MβCD on lipid body formation in SR-BI_{wt} and SR-BI_{red} macrophages. 3 independent experiments with ten images per experiment. (**e**) GP values at ruffles, PM and sub PM of CHO cells transfected with mSR-BI mutants (CDSRCD, SRCDSR). 35 – 60 cells per cell type. *P < 0.05, ***P < 0.001. Mann-Whitney Rank Sum Test (**c**) and Kruskal-Wallis One Way Analysis of Variants on Ranks (**e**). Data are representative of three different experiments (**a**, **b**, **c**).

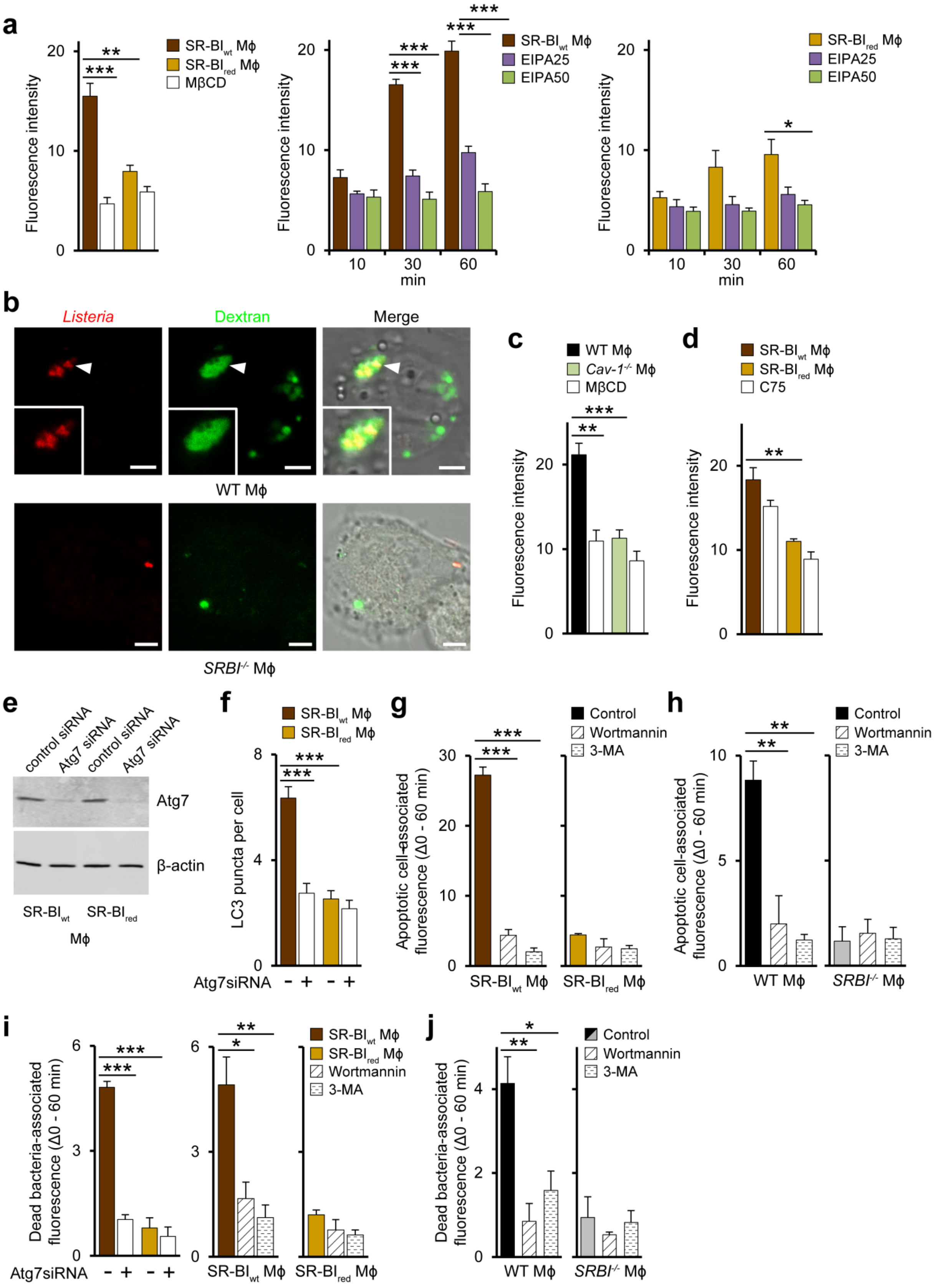

Supplementary Fig. 4| Role of host protein-induced membrane domains for cell entry of bacteria. **(a)** Internalization of labeled apoptotic neutrophils into human macrophages.

Influence of MβCD (left; 45 min) or EIPA (middle, right) on internalization into SR-BI_{wt} and SR-BI_{red} macrophages at indicated time points of incubation. n = 3, ten images per experiment. (**b**) Colocalization of internalized *Listeria* with dextran in WT macrophages but not *SRBI*$^{-/-}$ macrophages. 60 min incubation. Confocal microscopy images: Arrowheads indicate zoom region. Scale bar, 5 µm. Data are representative of three different experiments. (**c**) Internalization of labeled apoptotic neutrophils into WT and *Cav-1*$^{-/-}$ macrophages. Influence of MβCD. n = 3 (WT; control vs. MßCD) and n = 6 (*Cav-1*$^{-/-}$; control vs. MßCD), independent experiments with ten images per experiment. (**d**) Internalization of labeled apoptotic neutrophils into human macrophages. Effect of C75. n = 3, ten images per experiment. (**e**) SiRNA-mediated reduction of Atg7 expression in SR-BI_{wt} and SR-BI_{red} macrophages. (**f**) LC3 puncta in SR-BI_{wt} and SR-BI_{red} macrophages incubated with apoptotic neutrophils. Effect of reduction of Atg7 expression. n = 3, ten images per experiment. (**g**) Influence of wortmannin and 3-MA on degradation of engulfed apoptotic neutrophils in SR-BI_{wt} and SR-BI_{red} macrophages. n = 3, ten images per experiment. (**h**) Influence of wortmannin and 3-MA on degradation of engulfed apoptotic neutrophils in WT and *SRBI*$^{-/-}$ macrophages. n = 3, ten images per experiment. (**i**) Degradation of internalized dead bacteria in SR-BI_{wt} and SR-BI_{red} macrophages. Left: Effect of reduction of Atg7 expression. Middle and right: Influence of wortmannin and 3-MA. n = 3, ten images per experiment. (**j**) Effect of wortmannin and 3-MA on degradation of internalized dead bacteria in WT and *SRBI*$^{-/-}$ macrophages. n = 3, with ten images per experiment. *$P < 0.05$, **$P < 0.01$, ***$P < 0.01$. One-way ANOVA with Bonferroni post-test correction pairwise (**a**, **c**, **d**, **f**, **i** (left)) and versus control (**g**, **h**, **i** (middle, right), **j**).

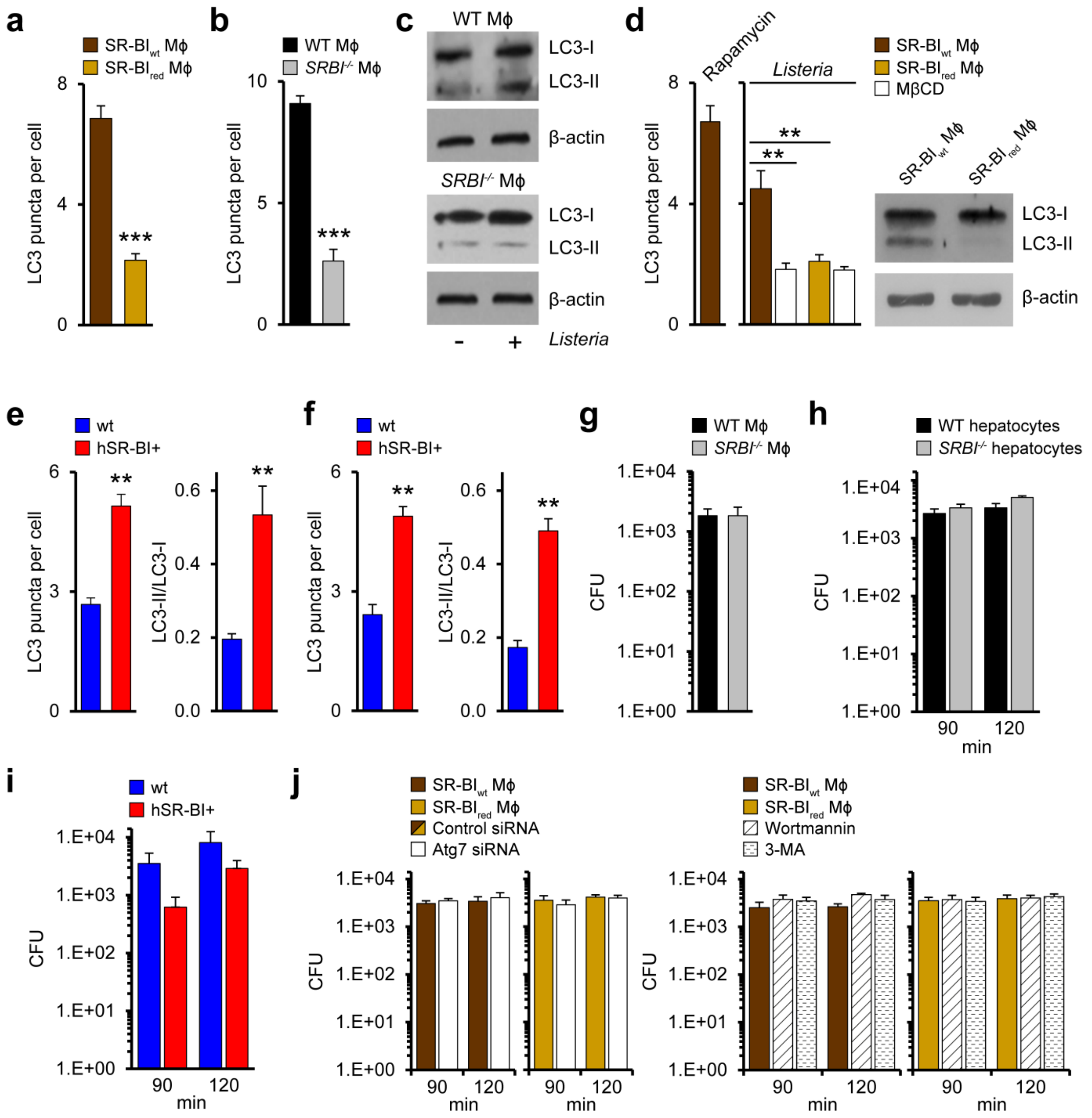

Supplementary Fig. 5| Autophagy-dependent elimination of host cell and bacterial corpses. (**a**) LC3 puncta in SR-BI$_{wt}$ and SR-BI$_{red}$ macrophages with engulfed dead bacteria. 60 min incubation. n = 3, with ten images per experiment. (**b**) LC3 puncta in WT and *SRBI*$^{-/-}$ macrophages with engulfed dead bacteria. 90 min incubation. n = 3, ten images per experiment. (**c**) Representative western blots (3 different experiments) of LC3-II formation in macrophages infected with Listeria. 60 min incubation. (**d**) LC3 puncta and LC3-II formation in *Listeria*-infected SR-BI$_{wt}$ and SR-BI$_{red}$ macrophages. Autophagy activation of control cells with rapamycin (5µM, 60 min, left). Influence of MβCD (right) after *Listeria* infection. 60

min incubation. n = 3, ten images per experiment. Representative western blots (3 different experiments, right). (**e**) LC3 puncta (left) and LC3-II formation (right) in *Listeria*-infected wt and hSR-BI+ cells. 75 – 90 min incubation. (**f**) LC3 puncta (left) and LC3-II formation (right) in *Yersinia*-infected wt and hSR-BI+ cells. 75 – 90 min incubation. (**g**) Intracellular survival of *Listeria* in macrophages. CFU values were measured after incubation with bacteria (60 min). n = 6. (**h**) Survival of *Listeria* in WT and $SRBI^{-/-}$ hepatocytes. Intracellular cfu measured at indicated time points after start of incubation of cells with *Listeria*. n = 6 (90min), n = 3 (120min). (**i**) Survival of *Listeria* in wt and hSR-BI+ cells. n = 3. (**j**) Effect of reduction in Atg7 expression (left) and of wortmannin or 3-MA (right) on cfu values in $SR\text{-}BI_{wt}$ and $SR\text{-}BI_{red}$ macrophages. n = 4 (control vs. Atg7 siRNA), n = 3 (control vs. wortmannin and 3-MA). **P < 0.01, ***P < 0.001. *t*-test (**a**, **b**, **e** (left), **f**), One-way ANOVA with Bonferroni post-test correction pairwise (**d**) and Mann-Whitney Rank Sum Test (**e**, right)

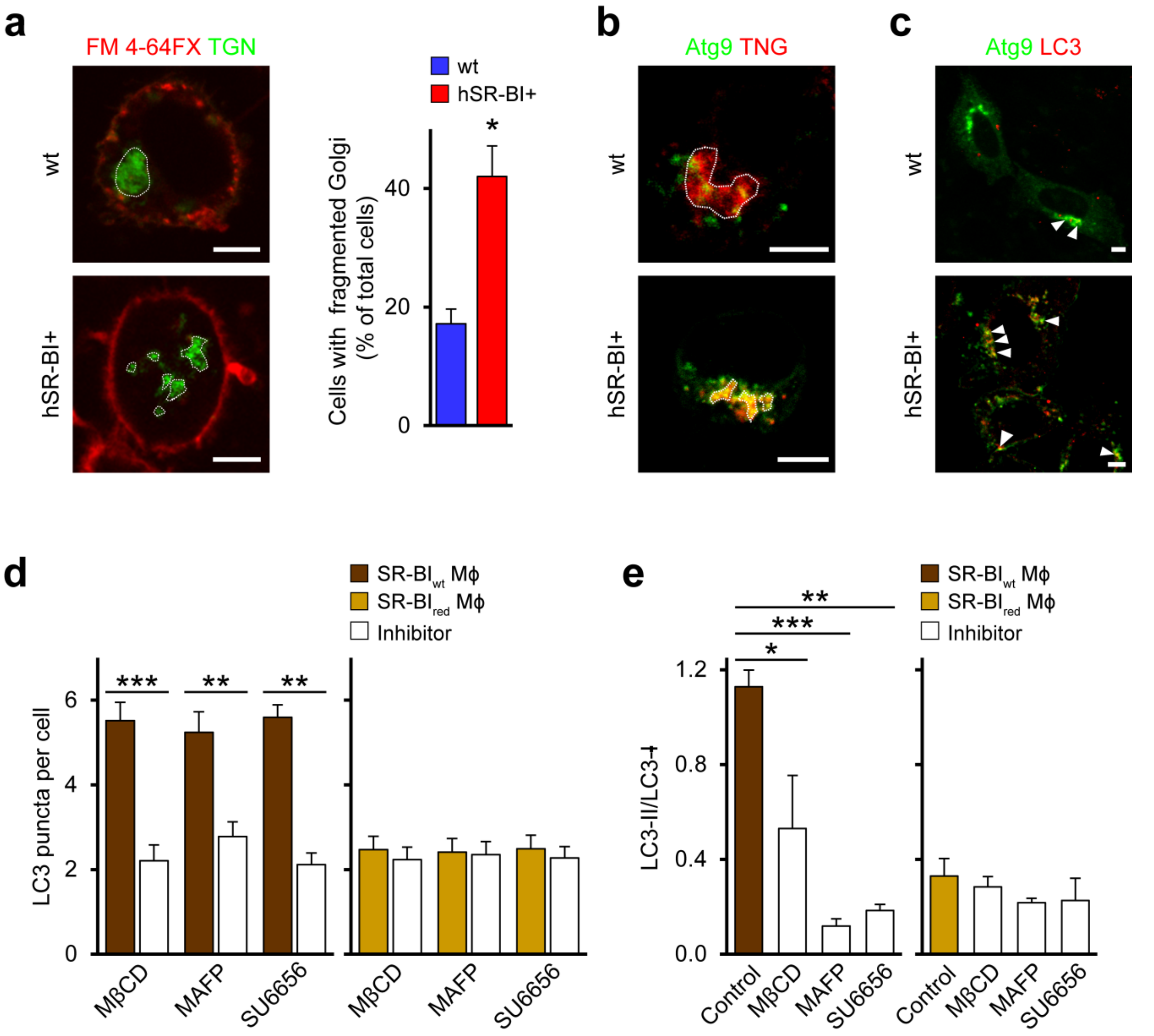

Supplementary Fig. 6| Cholesterol-induced Golgi fragmentation contributes to initiate autophagy. (**a**) Integrity of Golgi in wt and hSR-BI+ cells. Left: Red, plasma membrane; green, Golgi. Golgi structures delineated with dotted lines. Confocal microscopy images. Scale bar, 5 µm. Right: Percentage of cells with fragmented Golgi after incubation with *Listeria*. n = 3, ten images per experiment. (**b**) Association of Atg9 (green) with Golgi (red) in wt and hSR-BI+ cells after incubation with *Listeria*. Golgi structures delineated with dotted lines. Confocal microscopy images. Scale bar, 5 µm. (**c**) Colocalization of Atg9 (green) with LC3 (red) in wt and hSR-BI+ cells after incubation with *Listeria*. Confocal microscopy images. Arrowheads indicate colocalization. Scale bar, 5 µm. (**d**) Effect of MβCD and of inhibition of Golgi fragmentation with MAFP and SU6656 on LC3-puncta (n = 3, ten images

per experiment) and (**e**) LC3-I lipidation in SR-BI$_{wt}$ and SR-BI$_{red}$ macrophages incubated with apoptotic neutrophils (n = 3). *P < 0.05, **P < 0.01, ***P < 0.001. *t*-test (**a**) and One-way ANOVA with Bonferroni post-test correction pairwise (**d**) and versus control (**e**). Data are representative of three different experiments (**a**, **b**, **c**).

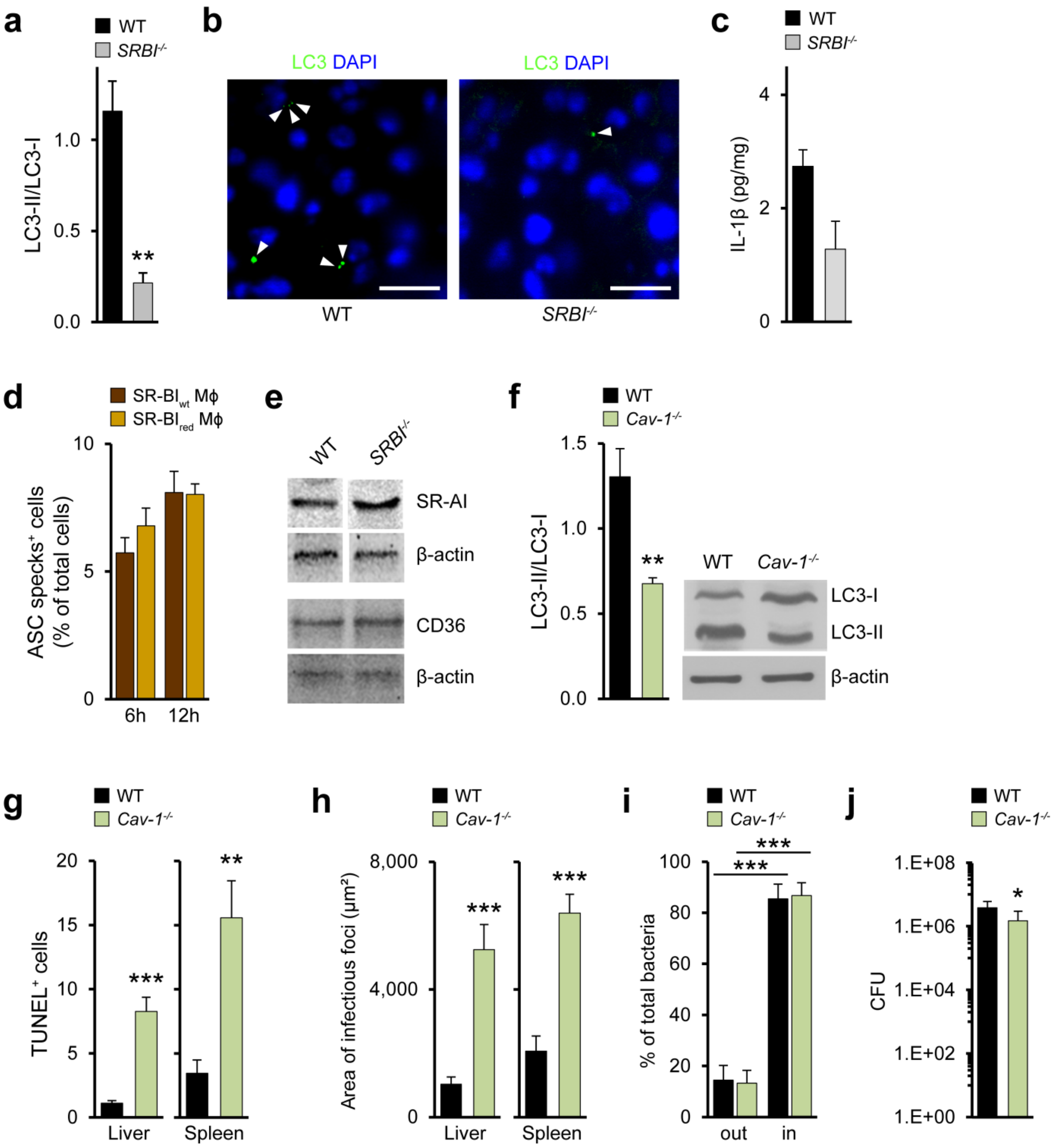

Supplementary Fig. 7| Cav-1-dependent autophagy activation limits organ damage. (**a**) LC3-II formation (expressed as LC3-II to LC3-I ratio) in livers of *Yersinia*-infected WT and *SRBI*$^{-/-}$ mice (d4). n = 4. (**b**) Confocal microscopy images of LC3 puncta in livers of *Yersinia*-

infected WT and *SRBI*$^{-/-}$ mice (d4). Scale bar, 20 µm. Data are representative of experiments on three different mice. (**c**) Liver levels of IL-1β in WT and *SRBI*$^{-/-}$ mice during *Listeria* infection (d3). n = 3 (WT), n = 5 (*SRBI*$^{-/-}$). (**d**) ASC specks in SR-BI$_{wt}$ and SR-BI$_{red}$ macrophages following a 6 and 12 h infection with *Listeria*. During the last 2 h of incubation with *Listeria* the cells were additionally exposed to apoptotic neutrophils. n = 3, ten images per experiment. (**e**) SR-AI and CD36 expression in livers of *Listeria*-infected WT and *SRBI*$^{-/-}$ mice (d3). Representative blots (2 different mice). (**f**) LC3-II to LC3-I ratio (left) in livers of *Listeria*-infected WT and *Cav-1*$^{-/-}$ mice (d3, n = 6). Right: Representative western blots (3 different mice) of LC3-II formation. (**g**) Number of TUNEL^{+} cells per visual field in *Listeria*-infected organs (d3) of WT and *Cav-1*$^{-/-}$ mice. n = 6 (WT, spleen), n = 7 (WT, liver) and n = 3 (*Cav-1*$^{-/-}$), 20 images per animal. (**h**) Area of infectious foci in *Listeria*-infected organs (d3) of WT and *Cav-1*$^{-/-}$ mice. n = 6 (WT) and n = 8 (*Cav-1*$^{-/-}$), five images per animal. (**i**) Distribution of *Listeria* inside and outside of infectious foci in WT and *Cav-1*$^{-/-}$ mice. n = 3, five images per animal. (**j**) Bacterial survival in *Listeria*-infected livers of WT and *Cav-1*$^{-/-}$ mice. n = 4 (WT), n = 8 (*Cav-1*$^{-/-}$). *P < 0.05, **P < 0.01, ***P < 0.01. *t*-test (**a**, **g**, **h** (spleen)), Mann-Whitney Rank Sum Test (**f**, **h** (liver), **j**), One-way ANOVA with Bonferroni post-test correction (**i**).

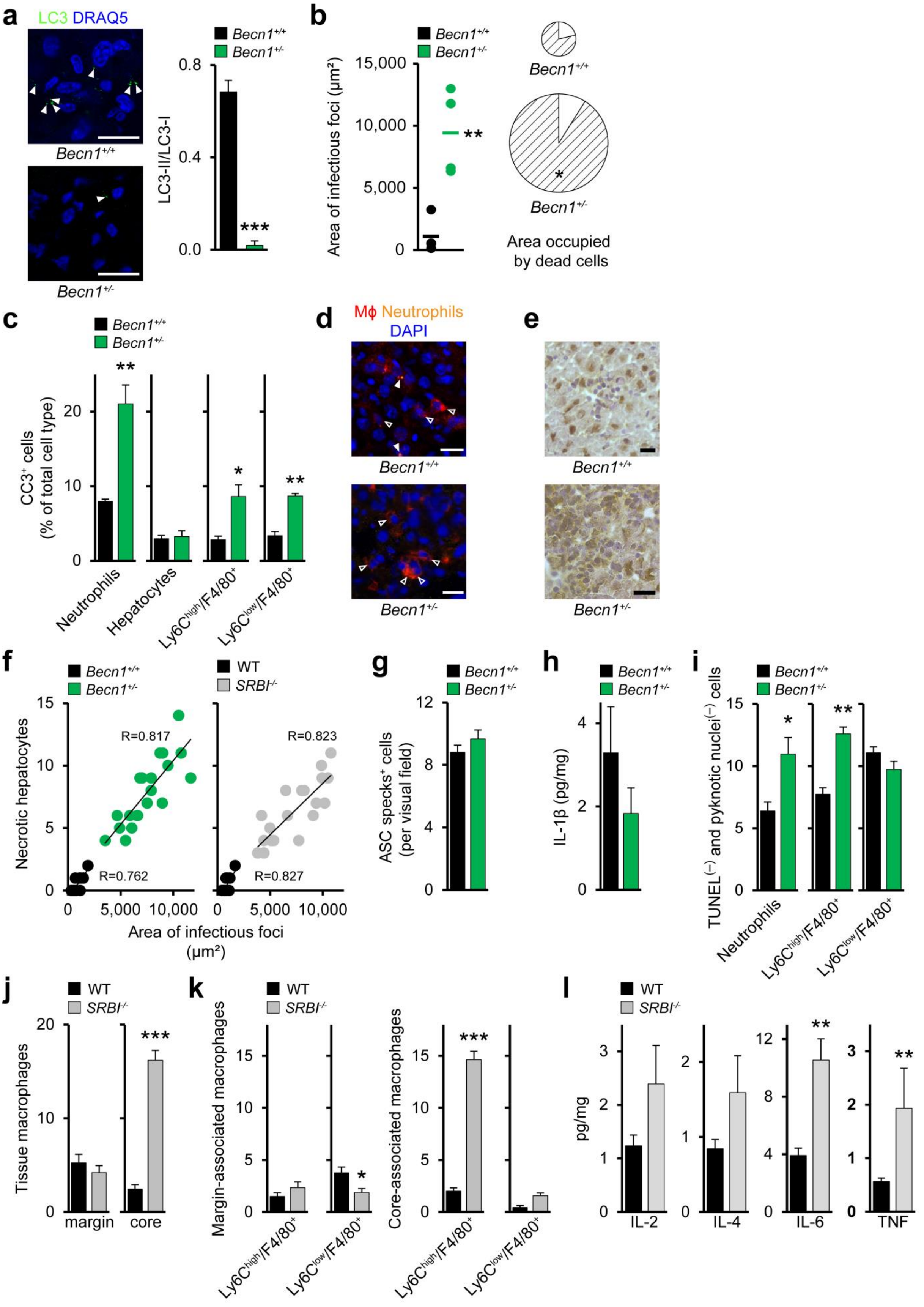

Supplementary Fig. 8| Autophagy inhibition reproduces liver damage characteristics in *SRBI*$^{-/-}$ mice. (**a**) LC3 puncta and LC3-II formation in livers of *Listeria*-infected *Becn1*$^{+/+}$ and

Becn1$^{+/-}$ mice (d3). Left: Confocal microscopy images. Scale bar, 20 µm. Right: Quantitative analysis of LC3-II formation (expressed as LC3-II to LC3-I ratio). n = 3. (**b**) Left: Area of infectious foci in *Listeria*-infected *Becn1*$^{+/+}$ and *Becn1*$^{+/-}$ mice (d3). n = 4, five to ten images per animal. Right: Quantitative analysis of foci area occupied by dead cells in *Listeria*-infected *Becn1*$^{+/+}$ and *Becn1*$^{+/-}$ mice. Shaded area shows area occupied by dead cells as percentage of total focus size. Significance symbol refers to the difference between the percentage of shaded area in *Becn1*$^{+/+}$ and *Becn1*$^{+/-}$ mice. n = 3, ten images per animal. (**c**) Quantification of cells positive for CC3^{+} in *Becn1*$^{+/+}$ and *Becn1*$^{+/-}$ animals as percentage of total cell type. n = 3, five images per animal. (**d**) Macrophages with internalized neutrophils in *Listeria*-infected livers of *Becn1*$^{+/+}$ and *Becn1*$^{+/-}$ mice (d3). Confocal microscopy images: Empty arrowheads, tissue macrophages; filled arrowheads, macrophages with internalized neutrophils. Scale bar, 20 µm. (**e**) Visualization of necrotic HNF4α^{+} hepatocytes in infectious foci of *Becn1*$^{+/+}$ and *Becn1*$^{+/-}$ livers at indicated days of infection. Fluorescence microscopy images. Scale bar, 20 µm. (**f**) Correlations between the area of infectious foci and the number of necrotic hepatocytes inside foci in *Becn1*$^{+/+}$ and *Becn1*$^{+/-}$ (left) as well as in WT and *SRBI*$^{-/-}$ (right) mice during *Listeria* infection. n = 15 (control), n = 20 (*Becn1*$^{+/-}$, *SRBI*$^{-/-}$). (**g**) Quantitative analysis of cells with ASC specks in livers of *Listeria*-infected *Becn1*$^{+/+}$ and *Becn1*$^{+/-}$ mice (d3). n = 3, five images per animal. (**h**) Tissue level of IL-1β in *Becn1*$^{+/+}$ and *Becn1*$^{+/-}$ mice during *Listeria* infection. n = 3 (*Becn1*$^{+/+}$), n = 4 (*Becn1*$^{+/-}$). (**i**) Number of cells lacking signs of cell death (cell marker^{+}, TUNEL^{-} , normal-sized nuclei) per visual field in *Listeria*-infected *Becn1*$^{+/+}$ and *Becn1*$^{+/-}$ mice. n = 3, ten images per animal. (**j**) Macrophage distribution in infectious foci in WT and *SRBI*$^{-/-}$ mice (d3). n = 3, ten images per animal. (**k**) Ly6C^{high} and Ly6C^{low} macrophages at margin and in core of infectious foci of *Listeria*-infected *Becn1*$^{+/+}$ and *Becn1*$^{+/-}$ mice (d3). n = 3, ten images per animal. (**l**) Cytokine levels in liver tissue of *Listeria*-infected WT and *SRBI*$^{-/-}$ mice. n = 6 (WT), n = 5 (*SRBI*$^{-/-}$). *P < 0.05, **P < 0.01, ***P < 0.001. *t*-test (**a**, **b**, **c**, **i**, **j**, **k**), Mann-Whitney Rank Sum Test (**l**), Linear

regression analysis (**f**). Data are representative of experiments on three different mice (**a**, **d**, **e**).
